# Regional brain morphometry in patients with traumatic brain injury based on acute- and chronic-phase magnetic resonance imaging

**DOI:** 10.1371/journal.pone.0188152

**Published:** 2017-11-28

**Authors:** Christian Ledig, Konstantinos Kamnitsas, Juha Koikkalainen, Jussi P. Posti, Riikka S. K. Takala, Ari Katila, Janek Frantzén, Henna Ala-Seppälä, Anna Kyllönen, Henna-Riikka Maanpää, Jussi Tallus, Jyrki Lötjönen, Ben Glocker, Olli Tenovuo, Daniel Rueckert

**Affiliations:** 1 Imperial College London, Department of Computing, London, United Kingdom; 2 Combinostics, Tampere, Finland; 3 VTT Technical Research Centre of Finland, Tampere, Finland; 4 Department of Clinical Medicine, University of Turku, Turku, Finland; 5 Division of Clinical Neurosciences, Turku Brain Injury Centre, Turku University Hospital, Turku, Finland; 6 Division of Clinical Neurosciences, Department of Neurosurgery, Turku University Hospital, Turku, Finland; 7 Perioperative Services, Intensive Care Medicine and Pain Management, Turku University Hospital and University of Turku, Turku, Finland; Center for Neuroscience and Regenerative Medicine, UNITED STATES

## Abstract

Traumatic brain injury (TBI) is caused by a sudden external force and can be very heterogeneous in its manifestation. In this work, we analyse T1-weighted magnetic resonance (MR) brain images that were prospectively acquired from patients who sustained mild to severe TBI. We investigate the potential of a recently proposed automatic segmentation method to support the outcome prediction of TBI. Specifically, we extract meaningful cross-sectional and longitudinal measurements from acute- and chronic-phase MR images. We calculate regional volume and asymmetry features at the acute/subacute stage of the injury (median: 19 days after injury), to predict the disability outcome of 67 patients at the chronic disease stage (median: 229 days after injury). Our results indicate that small structural volumes in the acute stage (e.g. of the hippocampus, accumbens, amygdala) can be strong predictors for unfavourable disease outcome. Further, group differences in atrophy are investigated. We find that patients with unfavourable outcome show increased atrophy. Among patients with severe disability outcome we observed a significantly higher mean reduction of cerebral white matter (3.1%) as compared to patients with low disability outcome (0.7%).

## 1 Introduction

With an estimated annual global incidence of 6.8 million cases, traumatic brain injury (TBI) imposes a significant burden on patients, their families, and health services [1, 2]. TBI is often referred to as the “silent epidemic” as symptoms, such as memory loss or cognitive deficits, tend to be less apparent [3]. Research findings on TBI obtained while doing sports [4] or in military conflicts [5] have increasingly brought the disease into the focus of the public [6]. Further, moderate and severe TBI are assumed to be an important risk-factor for dementias such as Alzheimer’s disease (AD) in late life [7–11].

TBI is typically caused by blunt force injury, penetrating injury, or blast injury and its pathology is dependent on the forces associated with the acceleration/deceleration event [12]. The pathological processes following the injury are highly complex and the exact mechanism causing functional impairment is not entirely understood [12, 13]. A common categorisation of disease-related processes distinguishes primary and secondary injuries [11, 12, 14]. Primary injuries often have a focal component caused by events such as the direct impact of an object hitting the head. Common consequences that should be differentiated include skull fractures, parenchymal contusions, haemorrhage and haematomas [11, 12, 15]. Intracranial haemorrhages and haematomas are the most common cause for rapid clinical deterioration and the complications are generally dependent on location and size of the haematoma [12]. Another injury mechanism, diffuse injury, is initiated by the strong accelerating or decelerating forces during the injury event. This is referred to as diffuse axonal injury (DAI) and assumed to be the predominant mechanism of TBI [11, 15–17]. TBI often results in both focal and diffuse injury which can evolve over time [12]. DAI is also a major determinant of disease outcome [14] and considered a long-lasting process that develops from focal axonal changes to slow axonal separation [18]. Next to the consequences of the primary head injury such as focal lesions, it is assumed that complex secondary pathophysiological processes continue damaging brain cells and thus influence the disease outcome [11, 15, 16, 19]. Neurodegenerative diseases and chronic inflammation can potentially be initiated by TBI and result in chronic neuronal damage [10, 11, 20]. A comprehensive description of TBI-related pathology and long-term secondary processes triggered by the injury event is given in Smith [12].

**Fig 1:**
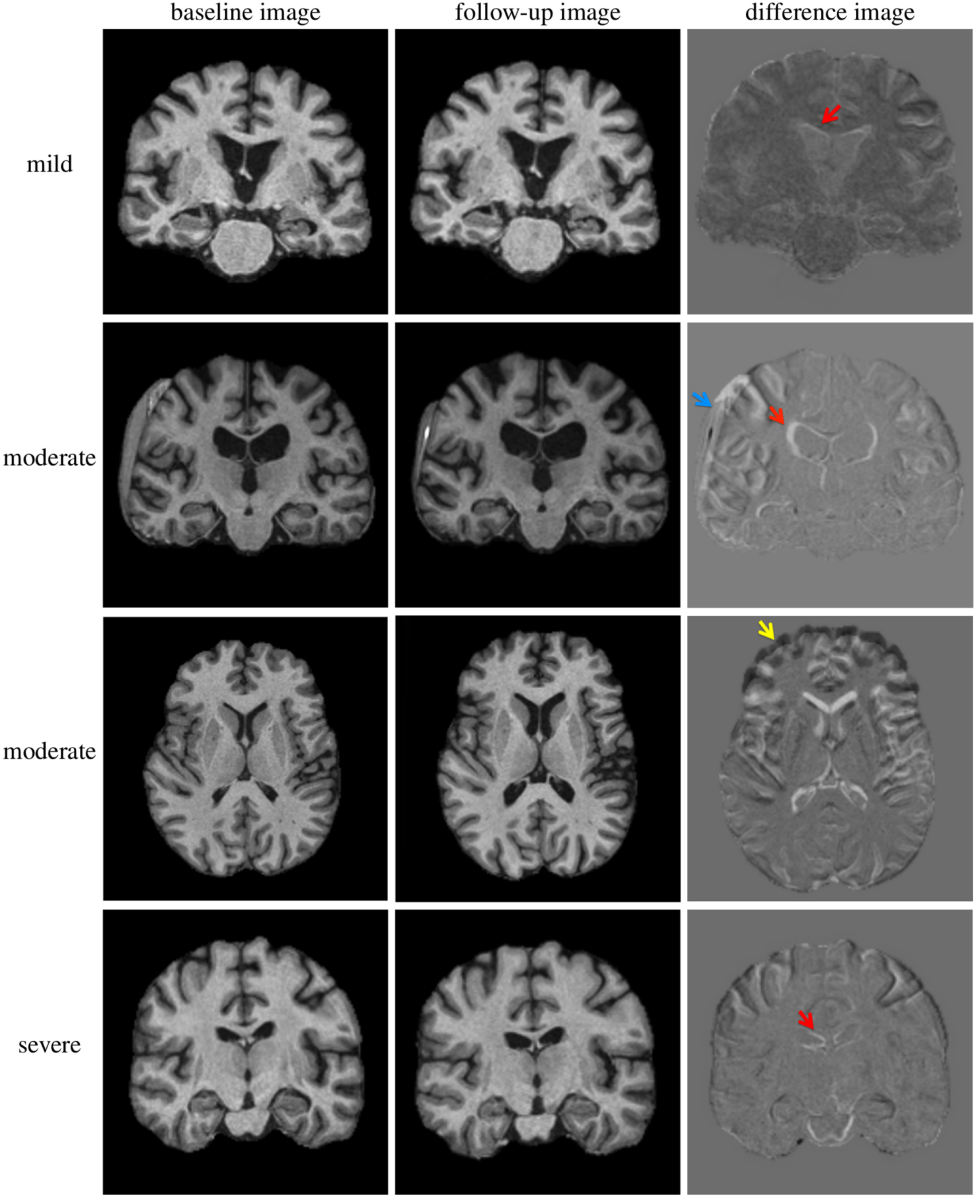
Examples of MR images of TBI patients. Four examples of T1-weighted MR images (brain extracted) of subjects from a prospective TBI cohort visualised in coronal or axial view. Top: patient with mild TBI (male, 72 years, Glasgow Coma Scale (GCS): 14, extended Glasgow Outcome Score (GOSe): 8, Marshall Classification Score (MCS): 1, cause: fall accident). Second row: patient with moderate TBI (female, 55 years, GCS: 3, GOSe: 4, MCS: 4, cause: fall accident). Third row: moderate TBI patient (male, 38 years, GCS: 11, GOSe: 5, MCS: n/a, cause: car accident). Bottom: patient with severe TBI (female, 33 years, GCS: 4, GOSe: 3, MCS: 2, cause: car accident). Left: baseline MR image acquired in the acute phase (days after the injury), Middle: follow-up MR image acquired in the chronic phase (months after the injury), Right: difference image of rigidly aligned images. Enlarged ventricles (red arrows), a subdural haematoma (blue arrow) and deformed/compressed frontal region (yellow arrow) are indicated in the difference images.

Evidence from neuroimaging such as magnetic resonance imaging (MRI) or computed tomography (CT) is often very subtle or completely absent, so that persistent symptoms tend to be explained e.g. through post-traumatic stress disorders or depression [17]. Four examples of subjects with TBI are visualised in Fig 1. These examples illustrate the heterogeneity of structural changes that can be subtle in both mild and severe TBI but also very apparent and variable. Although patterns of brain alteration have been shown to be predictors of outcome, such use of imaging data is mainly based on expert interpretation of visually inspected CT or MR images. In Maas et al. [21] the authors confirmed in a multivariate analysis the predictive value of individual characteristics quantified from CT for 6-months outcome prediction. In Jacobs et al. [22] it was shown for subjects with mild TBI that CT-based criteria are a valuable indicator to identify patients at risk of deterioration. However, they are only of limited value to predict the eventual outcome when compared to criteria such as patient age, alcohol intake or extra-cranial injuries [22].

**Fig 2:**
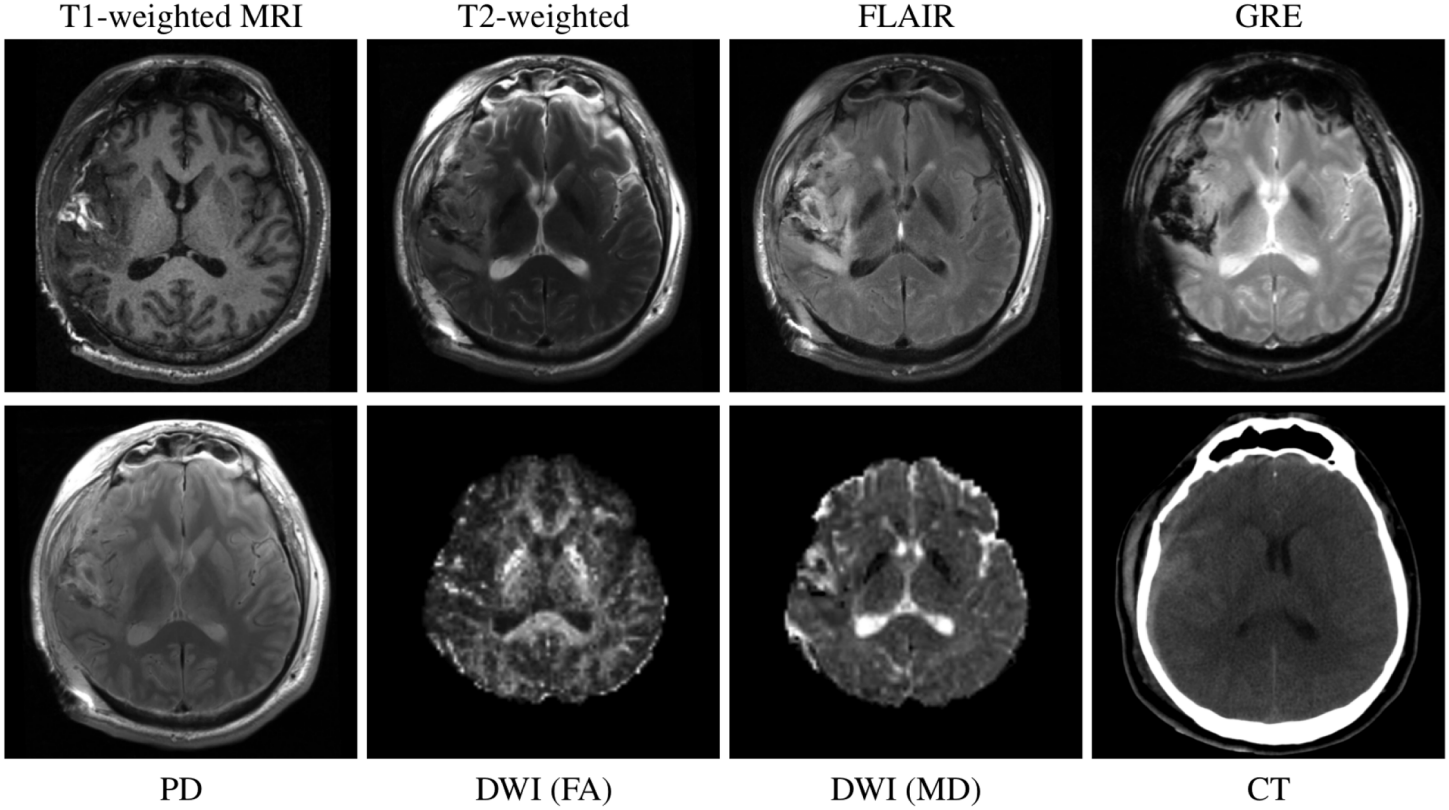
Brain images acquired with different imaging sequences/modalities. Images acquired from a patient with traumatic brain injury. The good tissue contrast in T1-weighted MR images and the pronounced contusions in the FLAIR sequence are apparent. Furthermore, gradient echo (GRE) and proton density (PD) weighted images are shown. Diffusion derived fraction anisotropy (FA) and mean diffusivity (MD) maps are also shown. CT is well suited to image bone injuries, oedema or intracranial bleeding. Note that this subject is from a different TBI dataset to the one used in this study. This study focuses on the analysis of T1-weighted images only, other sequences/modalities are shown to provide further background of MR imaging in TBI.

TBI is a very heterogeneous disorder and thus images from multiple modalities are required to characterise the disease [17, 23]. CT imaging is the modality of choice to identify skull fractures or other gross injuries that require immediate action [17]. CT provides critical information when treatment decisions at the acute TBI stage need to be made [21, 22]. However, contusions and lesions can be better assessed on fluid-attenuated inversion recovery (FLAIR) or gradient echo (GRE), especially on susceptibility weighted imaging (SWI), MR sequences. Next to this, T1-weighted (T1w) MRI provides good tissue contrast allowing the accurate segmentation of distinct anatomical structures. The definition of these regions of interest (ROI) is an important step towards a ROI-based analysis of information from diffusion weighted imaging (DWI) or functional imaging. Examples of typical imaging sequences acquired in the context of TBI are illustrated in Fig 2.

In this work we focus on structural T1-weighted MR images, in which subtle volumetric changes can be assessed. There is consensus that there is ongoing structural atrophy following TBI [24]. However, as these structural changes are difficult to assess visually on MRI or CT there is a high need for advanced methods that allow the quantification of atrophy [24]. The advancement of sensitive neuroimaging techniques is thus critical as it offers the potential to better understand, diagnose and treat TBI [2, 17, 24].

In contrast to other diseases of the human brain, such as AD, there is only a limited number of studies investigating the spatial distribution of structural changes in TBI [16, 25]. The need for accurate prognostic assessments was formulated already by Jennett et al. [26], however, prediction of TBI outcome remains a challenge. World-wide, TBI and the related processes are an understudied research area [15] and standard models to predict the outcome of TBI patients remain unavailable [2]. It is stated in Irimia et al. [2] that the combination of volumetric measures with brain connectivity/integrity measures from diffusion tensor imaging (DTI) (e.g. Bendlin et al. [25], Kinnunen et al. [27]) or with functional measures obtained through functional MRI (fMRI) (e.g. McAllister et al. [28], Sharp et al. [29], McDonald et al. [30]) might be key for future TBI research.

Accurate quantitative assessment of the neuroanatomic changes occurring during and after TBI is a difficult endeavour but crucial to assist the understanding of TBI disease progression. It is well known that a reduction of total brain volume and cerebral atrophy are common sequelae of TBI [19, 24, 25, 31, 32]. Recently, studies have found that next to this reduction in total brain volume several distinct structures such as amygdala, hippocampus or thalamus are involved in TBI [2, 13, 16, 20, 24, 33]. There is also increasing consensus that the volume of structural ROIs has the potential to support the outcome prediction of TBI [2, 13, 16, 24]. The early identification of affected brain regions that are likely to degenerate due to the primary injury could thus be key to an effective disease treatment [2]. Immediate and targeted treatment, enabled and supported by neuroimaging, could improve the final outcome of the disease but also reduce financial costs through shorter hospital stays [6]. In Bigler [24] the authors further pointed out that the volumetric measurement of subcortical structures might reveal irregularities that would be difficult to catch by visual inspection only.

It is becoming clear that the key to a better understanding of TBI disease progression is the combination of imaging information obtained from multiple modalities [2, 17, 23]. Here, the accurate delineation of anatomical ROIs is a critical prerequisite for subsequent ROI-based analyses of connectivity/function measurements. For example, in conjunction with DWI [13, 25] or positron emission tomography (PET) imaging [20], ROI-based analyses could enable a better understanding of the secondary processes that cause ongoing brain atrophy in the chronic phase of TBI.

Many of the conducted studies investigated group differences between patients with TBI and healthy subjects [16, 25, 34–36]. However, TBI is a very heterogeneous disease as it substantially depends on the type of injury (e.g. vehicle accident, fall, assault), severity of injury (e.g. vehicle speed, fall height, assault weapon) and location of the impact. It is due to this heterogeneity that comparably large sample sizes are required to show significant differences in research studies or treatment effects in clinical trials [2]. Methods to automatically extract biomarkers from brain MR images are thus a critical building block to enable large-scale TBI studies [2]. Measuring longitudinal change of the biomarkers is also hoped to enable a better understanding of the disease progression. For example, the causal relation of secondary degenerative processes to the primary injury is of high interest [2]. Nevertheless, current research on robust methods to automatically process MR images of injured brains is very limited [2, 6].

In the following an overview is given over studies that have investigated the potential of neuroimaging in the context of TBI. Particular focus is put on measures derived from structural MR brain images. A further summary can also be found in overview articles such as Bigler [24], Irimia et al. [2] or Shenton et al. [17].

### 1.1 Related work

Substantial group differences in grey matter (GM) density between healthy control subjects and patients with TBI were confirmed using voxel-based morphometry (VBM) [32, 34, 37]. Salmond et al. [34] performed VBM to compare MR images from 22 patients acquired at least six months post injury to a matched set of control subjects. In this study reduced GM density in thalamus, basal forebrain, hippocampal formation and regions of the neocortex were identified [34]. In Gale et al. [32], the authors employed VBM to compare the GM density of nine patients with TBI (mild to severe injury) to age and gender matched controls. Based on follow-up MR images acquired around one year after the injury, the authors found a significant decrease in GM concentration in a multitude of brain regions including the cerebellum, frontal and temporal cortices, but also subcortical structures [32]. In Kim et al. [35] a study cohort of 29 patients with at least moderate TBI and 20 healthy control subjects was analysed using tensor-based morphometry (TBM). Local reductions in WM and subcortical regions such as thalamus, corpus callosum and caudate were shown in the TBI group.

Most of the few existing studies that analysed structural morphometric measures [13, 16, 33] were based on the segmentation techniques available in FreeSurfer (http://surfer.nmr.mgh.harvard.edu/, last accessed 09 November 2017, [38–40]) and have investigated small patient cohorts [13, 16]. Warner et al. [13] analysed the relation of axonal injury quantified from DTI with structural volumes in the chronic phase (8 months after injury) of the injury. Structural volumes of hippocampus, amygdala and thalamus but also of cortical ROIs were stronger correlated with white matter (WM) integrity at the chronic than at the acute time point. This suggests that white matter integrity can change due to secondary processes far beyond the acute phase [13]. In Strangman et al. [33], 50 patients that sustained TBI were enrolled in a memory rehabilitation program and their individual progress recorded. The study investigated the predictive value of structural brain volumes with respect to the outcome of the rehabilitation. Ramlackhansingh et al. [20] used ROIs segmented from structural MRI and PET to demonstrate that inflammatory processes remain active for months or years following a brain trauma. Several studies [13, 16, 20, 33] have identified structures, including thalamus and hippocampus that are affected by TBI and are of significant value when predicting clinical outcome.

Longitudinal changes following the injury event have also been analysed [6, 16, 25, 36]. Bendlin et al. [25] performed a longitudinal analysis of 46 patients with TBI with respect to 36 matched healthy controls using VBM. Both structural integrity quantified from DTI and WM/GM density calculated from T1w images declined while scores related to neuropsychological function improved. In Sidaros et al. [36] longitudinal changes in the months following a severe TBI were investigated. In this study, 24 patients were compared to 14 healthy subjects. The authors found both an increased reduction in brain volume when compared to the healthy control group and regional involvement of brainstem, thalamus, corpus callosum, putamen and cerebellum. In Warner et al. [16] the authors analysed the correlation of structural brain atrophy of 25 patients who had sustained a DAI with functional outcome. Several brain structures showed significantly increased structural atrophy when compared to a control group of 22 age and gender-matched controls eight months post injury. Irimia et al. [6] compared TBI related changes, as assessed from images acquired with multiple MR sequences in three representative patients. The authors demonstrated how semi-automatic methods can support patient monitoring, damage assessment and quantification of temporal changes in clinical practice. Wang et al. [41–43] developed such a semi-automatic method to estimate subject-specific atlases for the segmentation of longitudinal MR data.

The automatic structural segmentation of MR brain images of patients with TBI remains, however, a difficult endeavour as most existing methods lack robustness towards TBI-related changes in anatomy [2, 6]. In the presence of gross pathologies such as hemorrhagic lesions/oedema (in the acute phase) or substantial atrophy (in the chronic phase) most of the established segmentation techniques yield unsatisfying results. While in many neurodegenerative diseases, such as AD, brain changes are consistent with disease progression, MR brain images of patients with TBI can show inconsistent and gross pathological change. It is this high variability and extent of brain change following a moderate or severe TBI that makes the segmentation task so demanding. A further discussion of current and potential future research directions is provided in Irimia et al. [2] and Shenton et al. [17].

### 1.2 Contribution and overview

In this work we employ a fully-automatic segmentation method to quantify biomarkers based on structural volume and structural asymmetry of 67 patients who sustained mild to severe traumatic brain injury. We analyse the potential value of these biomarkers that are automatically extracted at the acute injury stage to predict the outcome severity of the injury. We quantify structural atrophy occurring between the acute and chronic disease stage and find that patients with poor outcome suffer from increased brain atrophy.

The manuscript is organised as follows: First, we describe prospectively acquired study data that we aim to analyse in this work in Section 2. The applied approaches for feature (biomarker) extraction and classification are then described in Section 3. In Section 4.1, a cross-sectional analysis at the acute stage of the injury explores whether individual structural biomarkers have potential to predict patient-specific injury outcome. Further, a longitudinal analysis is performed in Section 4.2 and structural atrophy rates are calculated between images acquired at the acute and chronic disease stage. Group differences are investigated between patient groups of distinct outcome categories. In Section 5 the presented findings and limitations of the approach are discussed. Further, segmentation examples are provided that were obtained on brain MR images with disease related changes such as for example subdural haematomas, substantial structural deformation or atrophy. Section 6 concludes this manuscript.

## 2 Materials

The imaging data was acquired at Turku University Hospital, Finland in the course of the TBIcare project (http://www.tbicare.eu, last accessed 09 November 2017). For the T1w MR images an MPRAGE sequence was acquired on a Siemens Verio 3T system with the following parameters: TR 2300 ms, TE 2.98 ms, TI 900 ms, flip angle 9°, matrix size 256 × 249 × 176 and an isotropic voxel size of 1.0mm × 1.0mm × 1.0 mm, sagittal slices, using Prescan Normalizer, 2D distortion correction and a standard 12 channel head coil.

Over the course of the project a total of 141 subjects with mild to severe TBI have had MR images taken both at the acute stage of the injury (baseline) and in the chronic phase (follow-up) of the disease. Following the definition used in Newcombe et al. [44], the baseline images in this study were taken either in the acute or subacute phase. For readability both stages will be referred to as ‘acute’. All study subjects gave their informed consent for participating in the study, and the study protocol was accepted by the Ethical Committee of the Hospital District of Southwest Finland. Specifically, a written informed consent was obtained from all subjects, or where the subject remained unable to give the consent, from the proxy. The study was approved by the Ethical Committee of the Hospital District of Southwest Finland. In total 120 subjects were processed for which both baseline and follow-up images were available when the analysis was started. After visual review, six subjects were excluded due to low image quality or errors in the data description.

Characteristics of the remaining 114 patients are summarised in Table 1. Those 114 patients are further reduced to 67 study subjects in order to obtain age-matched patient groups. This is further described in Section 2.2.

**Table 1:** Overview of all processed MR images. Table shows patient gender, patient age, scan time relative to injury, GCS, MCS and TBI severity.

GOSe	low disability	moderate disability	severe disability
	7 & 8	5 & 6	3 & 4
# of subjects	69	32	13
gender (# male / # female)	47/22	21/11	4/9
years of age (median [min; max])	40 [18; 82]	51 [19; 83]	74 [33; 86]
days since injury, acute scan (median [min; max])	14 [1; 50]	22 [1; 51]	22 [4; 51]
days since injury, chronic scan (median [min; max])	230 [151; 399]	228 [177; 429]	251 [180; 422]
Glasgow Coma Scale (median [min; max])	15 [3; 15]	15 [3; 15]	14 [3; 15]
Marshall score (median [min; max])	1 [1; 5]	2 [1; 5]	5 [2; 5]
TBI severity^†^ (median [min; max])	2 [1; 4]	2.5 [2; 5]	3 [2; 5]

^†^: 1: very mild, 2: mild, 3: moderate, 4: severe, 5: very severe

### 2.1 Clinical information

In addition to MR imaging data the clinical variables age, gender, Glasgow Coma Scale (GCS), Marshall Classification Score (MCS) [45], extended Glasgow Outcome Score (GOSe) [26, 46] and TBI severity were available. The GCS is a clinical score that quantifies a patient’s level of consciousness at the acute stage of the injury [47]. The GCS is the most common criteria to determine the severity (e.g. mild, moderate, severe) of a brain injury in the acute setting [12, 48–50]. After the head injury the GCS is potentially assessed several times, e.g. at the injury site before pre-hospital care, at hospital admission and in the intensive care unit. The GCS might not have been recorded for each patient at each time point. Thus a pragmatic approach was followed and the GCS score chosen that was recorded first. This is usually either at the injury site or at admission to the hospital. MCS is also assessed at the acute stage [45], a score based on the worst acute CT image within 24 hours of injury. MCS takes into account brain pathology such as lesion load or the presence of oedema and midline shift caused by the injury. The MCS groups 5 and 6 were pooled together, which means that the MCS scores did not distinguish between evacuated and non-evacuated mass lesions. Further, the GOSe score was assessed on the day when the follow-up MR image was acquired. The GOSe groups 3 & 4, 5 & 6 and 7 & 8 are summarised into three patient groups with severe, moderate and low disability outcome respectively. This was necessary to obtain reasonable group sizes and corresponds to using the GOS five point scale instead of the eight point GOSe. More details on the MCS and GOSe groups is provided in S1 File. TBI severity was classified based on combining GCS and the duration of post-traumatic amnesia (PTA), whichever gave a more severe index [49, 50]. Very mild = GCS 15 and PTA less than 1 hour; mild GCS 13-15 and PTA < 24 hrs, moderate GCS 9-12 or PTA > 24 hrs but less than one week; severe GCS 3-8 or PTA > 1 week; very severe PTA > 4 weeks.

### 2.2 Age-matching of patient groups

**Fig 3:**
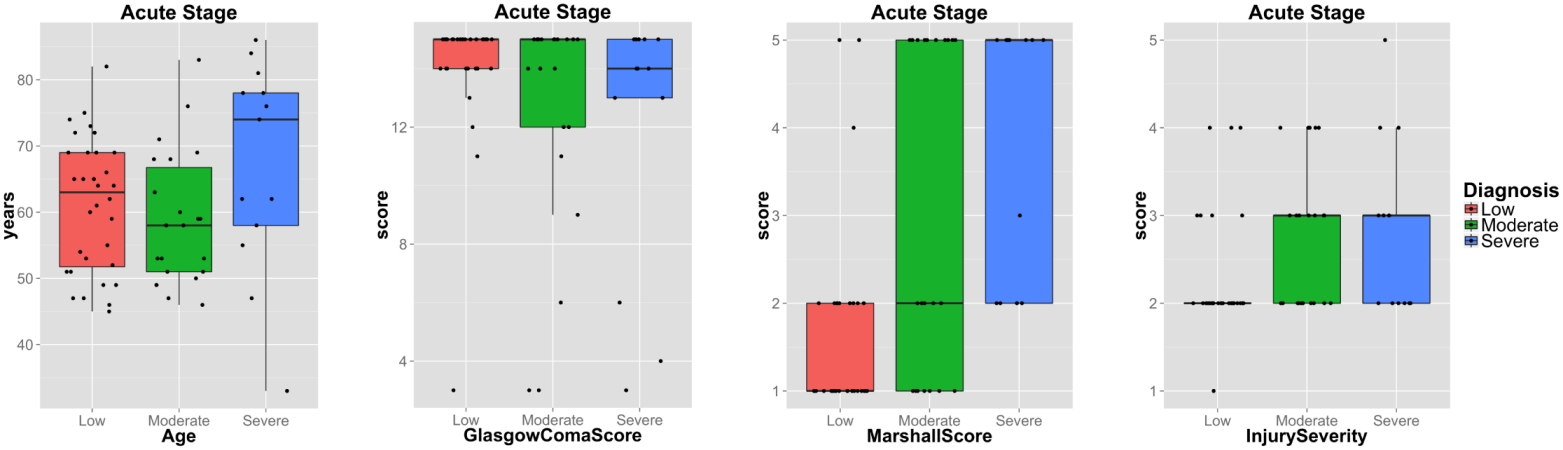
Boxplots of clinical variables. Plots of age, GCS, MCS and TBI severity of patients with low, moderate or severe disability outcome. Shown are only subjects of the age-matched dataset. Boxplots were created with the ggplot2 package of R (http://docs.ggplot2.org/0.9.3/geom_boxplot.html, last accessed: 09 November 2017). The plots show the median, 25%/75% quantiles (hinges), smallest/largest observation greater/less than or equal to lower/upper hinge -/+ 1.5*IQR (IQR: interquartile range). Data points were jittered along x-axis for better visualisation.

The study groups which are summarised in Table 1 show a significant mismatch in age (p < 0.001 for all groups with respect to the severe outcome group). It is important to account for these substantial differences when studying changes caused by the disease to minimise age-related effects. Due to the limited size of the study cohort this is a challenging endeavour. The group with severe disability outcome has the fewest samples and subjects are significantly older in age. In order to not further reduce the size of this group, all subjects were removed from the low and moderate disability outcome groups that are younger than 45 years of age. The age difference between the groups was no longer significant (p > 0.05) after this correction. This approach improved the age-match between study groups, however, reduced the number of study subjects from 114 to 67. An overview over the remaining subjects that will be studied in the following is provided in Table 2. The distribution of age, GCS and MCS is illustrated with respect to the three outcome groups in Fig 3.

**Table 2:** Overview of the data used for the analysis. Table shows patient gender, patient age, scan time relative to injury, GCS, MCS and injury severity. Study groups were age-matched by removing patients with low and moderate outcome disability that were younger than 45 years of age. Significant group differences (two-sided unpaired Student’s t-test) with respect to the low disability outcome group are indicated with *l* (p < 0.05) and *L* (p < 0.01). There are no significant differences between the moderate and severe disability outcome group.

GOSe	low disability	moderate disability	severe disability
	7 & 8	5 & 6	3 & 4
# of subjects	32	22	13
gender (# male / # female)	21/11	14/8	4/9^l^
years of age (median [min; max])	63 [45; 82]	58 [46; 83]	74 [33; 86]
days since injury, acute scan (median [min; max])	15 [1; 50]	23 [2; 51]	22 [4; 51]
days since injury, chronic scan (median [min; max])	225 [151; 276]	227 [177; 429]	251 [180; 422]^l^
Glasgow Coma Scale (median [min; max])	15 [3; 15]	15 [3; 15]	14 [3; 15]^l^
Marshall score (median [min; max])	1 [1; 5]	2 [1; 5]^*L*^	5 [2; 5]^*L*^
TBI severity^†^ (median [min; max])	2 [1; 4]	3 [2; 4]^l^	3 [2; 5]^l^

^†^: 1: very mild, 2: mild, 3: moderate, 4: severe, 5: very severe

*l*, *L*: significant different to low disability outcome group (p < 0.05, p < 0.01)

## 3 Methods

### 3.1 Cross-sectional and longitudinal structural segmentation

All available images were preprocessed by first correcting for intensity inhomogeneities with the N4 bias correction algorithm [51]. Images were subsequently brain extracted using pincram [52], an iterative, atlas-based method that was developed with particular focus on robustness. Each image was then segmented individually using Multi-Atlas Label Propagation with Expectation-Maximisation based refinement (MALPEM) as described in [53]. As brain atlases, the 30 manually annotated Neuromorphometrics (NMM) brain atlases were employed. Those atlases were provided by Neuromorphometrics, Inc. under academic subscription (http://Neuromorphometrics.com/, last accessed: 09 November 2017). The atlases distinguish between 40 non-cortical and 98 cortical brain regions. A complete list of all individual regions is provided in S1 File. In MALPEM, the 30 manually annotated brain atlases are propagated to the image that is to be segmented based on transformations calculated with the robust registration approach MAPER [54, 55]. The propagated atlases are subsequently fused into a consensus probabilistic prior estimate using a locally weighted fusion approach based on the Gaussian-weighted sum of squared distances (GSSD) [53]. The GSSD is calculated on images that were intensity normalised using a robust linear rescaling approach [56, 57]. The probabilistic prior estimate is refined to both improve segmentation accuracy and account for pathology in the images optimising an intensity-based Gaussian mixture model with an Expectation-Maximisation approach. A modified version of MALPEM that does not rely on MAPER is publicly available at: https://github.com/ledigchr/MALPEM (last accessed: 09 November 2017).

**Fig 4:**
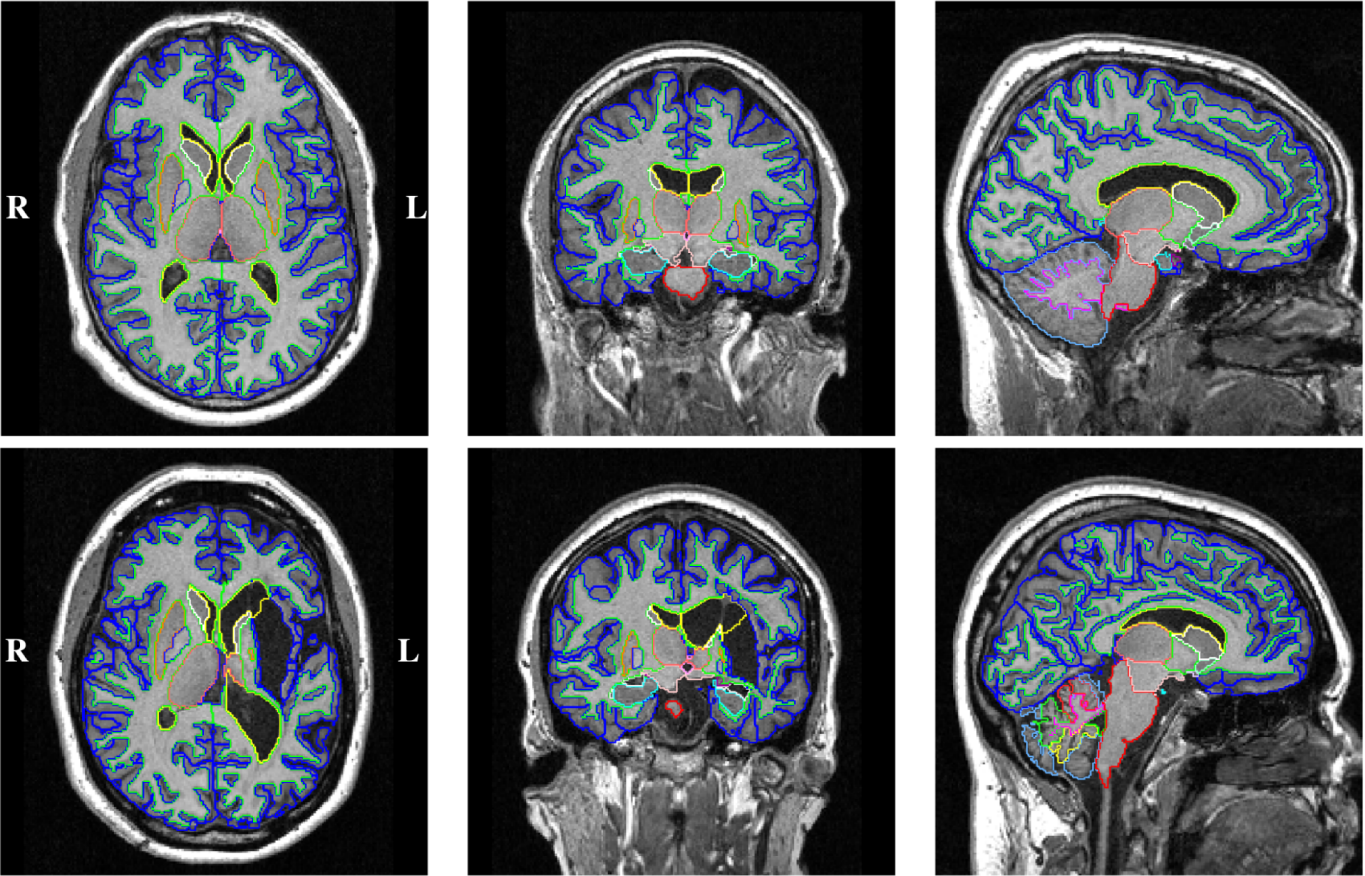
Example cross-sectional segmentation results. Results of images acquired at the acute stage of a TBI. Top: TBI010, male, 21 years of age with mild TBI (GCS: 15) caused by a fall accident, favourable disease outcome (GOSe: 8), no visible intracranial pathological changes on CT (MCS: 1), image acquired 2 days after injury. Bottom: TBI038, female, 47 years of age with mild TBI (GCS: 15) caused by a fall, unfavourable disease outcome (GOSe: 4), substantial pathological changes on CT (MCS: 5), image acquired 4 days after injury with clear sequelae of intra-cerebral haematoma. Before the actual TBI event this patient suffered a spontaneous intra-cerebral haematoma due to an untreated hypertension. The colour scheme is described in S1 File.

The refined, time-point specific probabilistic segmentation output and the intensity normalised, brain extracted images are then employed to perform the consistent longitudinal segmentation as described in [58] (MALPEM4D). We thus used a symmetric affine registration approach to align the subject-specific probabilistic priors of individual time-points and intensity-normalised images to a common intermediate space [59, 60]. To account for remaining differential bias between intra-patient acquisitions in the presence of disease-related pathology we employ the spatially weighted correction approach proposed in Ledig et al. [58]. MALPEM4D is an approach that employs spatially and temporally varying coupling weights between time-points to obtain temporally consistent segmentation estimates. In the context of TBI, gross structural changes can be expected between both imaging time points. Thus a weighted differential bias field correction procedure was used [58]. All brain masks and segmentation results were visually reviewed to ensure reasonable accuracy in the presence of pathology. Two typical segmentation results are visualised in Fig 4.

### 3.2 Feature extraction and classification setup

Group differences were investigated between GOS groups. Further, classification experiments were performed to quantify the accuracy of predicting the GOS outcome category when using automatically calculated features based on imaging data available at the acute disease stage. In this work we use the term “feature” for a single measured biomarker (e.g. structural volume, asymmetry, atrophy) or clinical variable (e.g. gender, age, Marshall score).

All 67 subjects were analysed cross-sectionally at the *acute* stage of the TBI and longitudinally employing the follow-up image acquired in the *chronic* phase of the disease. All available non-cortical structural volumes were used as features. Since corresponding volumes in the left and right brain hemisphere were merged we consider 21 non-cortical features (28/2+7 = 21, c.f. S1 File). Note that the seven non-cortical structures 3rd and 4th ventricles, brain stem, cerebrospinal fluid (CSF), cerebellar vermal lobule I-V, cerebellar vermal lobule VI-VII and cerebellar vermal lobule VIII-X have no symmetric counterpart. Individual structural volumes were summarised (added up) into surrogate structures: ventricles, cortical GM, deep GM, WM, brain tissue (BrainTissue) and total brain volume (Brain) (6 features). Note that the difference between BrainTissue and Brain is the exclusion/inclusion of ventricular/CSF volume respectively. The structures cerebral exterior, vessel and optic chiasm were excluded from the analysis due to their very small size. Due to the limited number of study subjects and the heterogeneity of the injury, cortical structures were only investigated as surrogate structure (cortical GM) and not considered as individual features. A full list of all individually segmented structures and how they contribute to surrogate structures is provided in S1 File.

In the *cross-sectional* analysis at the acute stage, structural asymmetry was quantified as the absolute asymmetry index (AAI) [53, 61, 62] based on a structure’s volume (V) in the left and right hemisphere, which is defined as:

$$\begin{array}{c} \text{AAI}=100\%\frac{|V_{\text{left}}-V_{\text{right}}|}{0.5(V_{\text{left}}+V_{\text{right}})} \end{array}$$ (1)

The AAI was calculated for the 14 non-cortical structures appearing in both brain hemispheres and the six surrogate structures. Additionally the AAIs of all individual non-cortical, cortical and all brain structures were added up. Note that the sum of, for example, all cortical AAIs is different to the AAI of the cortical GM surrogate structure. The segmentations at the acute stage were calculated with MALPEM and not MALPEM4D. This is important since MALPEM4D exploits information of later scanning time points, which is not yet available at the acute disease stage. We therefore ensured that when predicting outcome disability during the acute stage indeed only information that is available during the early disease stage is employed. Further, we incorporated into our analysis the five clinical features: age, gender, GCS, MCS and TBI severity. A detailed list of all classification features that were used for the cross-sectional analysis is provided in Table 3.

**Table 3:** Overview of all considered features.

**Cross-sectional**	number of features	names of features
clinical features (clinical)	5	age, gender, GlasgowComaScore, MarshallScore, InjurySeverity
volumetric (MALPEM^vol^)	27	volume of individual brain structures: AccA, Am, Cau, CblmExt, CblmWM, CrblWM, Hc, infLV, LV, Pa, Pu, Th, vDC, BF, 3rdVent, 4thVent, BS, CSF, CVL1t5, CVL6t7, CVL8t10 (21) volume of surrogate classes: DeepGreyMatter, CorticalGreyMatter, WhiteMatter, Ventricles, Brain, BrainTissue (6)
asymmetry (MALPEM^sym^)	23	asymmetry of the 14 brain structures: AccA, Am, Cau, CblmExt, CblmWM, CrblWM, Hc, infLV, LV, Pa, Pu, Th, vDC, BF (14) asymmetry of surrogates: DeepGreyMatter, CorticalGreyMatter, WhiteMatter, Ventricles, Brain, BrainTissue (6) accumulated asymmetry of all cortical, all non-cortical and all structures (3)
**Longitudinal**	number of features	names of features
clinical features (clinical)	2	age, gender
volumetric (MALPEM4D^vol^)	27	volume change of individual brain structures: AccA, Am, Cau, CblmExt, CblmWM, CrblWM, Hc, infLV, LV, Pa, Pu, Th, vDC, BF, 3rdVent, 4thVent, BS, CSF, CVL1t5, CVL6t7, CVL8t10 (21) volume change of surrogate classes: DeepGreyMatter, CorticalGreyMatter, WhiteMatter, Ventricles, Brain, BrainTissue (6)

For the *longitudinal* analysis, structural volumes of all 67 subjects were extracted based on their MALPEM4D segmentations. Atrophy rates were calculated using the logarithmic transform as *Δ*_*v*_(*t*^1^, *t*^2^) = 100%ln(*v*_*t*^2^_/*v*_*t*^1^_). Note that atrophy rate and volume change is used interchangeable, which means that a *positive* atrophy rate indicates an *increase* in volume. The volume change was measured for the six surrogate structures and for the individual 21 non-cortical structures. This yields, considering age and gender, 29 features for the longitudinal analysis. Even though the analysis was limited to changes in structural volume, other longitudinal alterations such as changes in brain symmetry could be investigated. A detailed list of all classification features that were used for the longitudinal analysis is provided in Table 3.

For classification 100 runs of a 6-fold cross-validation (CV) were performed using linear discriminant analysis (LDA) for individual features and support vector machine (SVM) or random forest (RF) classifiers when combining multiple features. All classifiers are trained to discriminate between two disease severity categories (e.g. low disability vs. severe disability outcome). A classification framework was implemented using MATLAB that relies on classify (LDA), TreeBagger (RF, 100 trees) and libSVM (linear SVM, [63]). The features were normalised (rescaling) individually based on the respective training set to the range 0 to 1 for the SVM classification. Both the LDA classifier, which was used for single-feature classification only, and the RF classifier do not require feature normalisation. No explicit correction for age, gender or head size was applied. This is further discussed in Section 5. Next to standard classification accuracy (ACC), we also quantified the balanced classification accuracy (bACC, [64]) to account for imbalanced group sizes. The bACC is calculated as the arithmetic mean of sensitivity (SENS) and specificity (SPEC).

Significance levels were quantified as p-values of two-sided, unpaired Student’s t-tests. To account for multiple comparison we also calculate Bonferroni corrected significance levels. Further, effect sizes were calculated as *Cohen’s d* by dividing the differences of the sample means (absolute value) by their pooled standard deviation [65–67]. According to Cohen [65] an effect size of d = 0.2 can be considered as small, of d = 0.5 as medium and of d = 0.8 as large. Reporting the effect size in addition to the p-value is important as it quantifies the magnitude of a group difference, while a low p-value only confirms its existence [68].

## 4 Results

### 4.1 Cross-sectional analysis

Individual brain ROIs were extracted from the *acute* T1w MR images. Features (volume, asymmetry) were derived from these ROIs and their potential investigated to discriminate TBI patients according to their *outcome* severity. Example segmentation results of a TBI patient with low disability outcome and a patient with severe disability outcome are shown in Fig 4.

**Fig 5:**
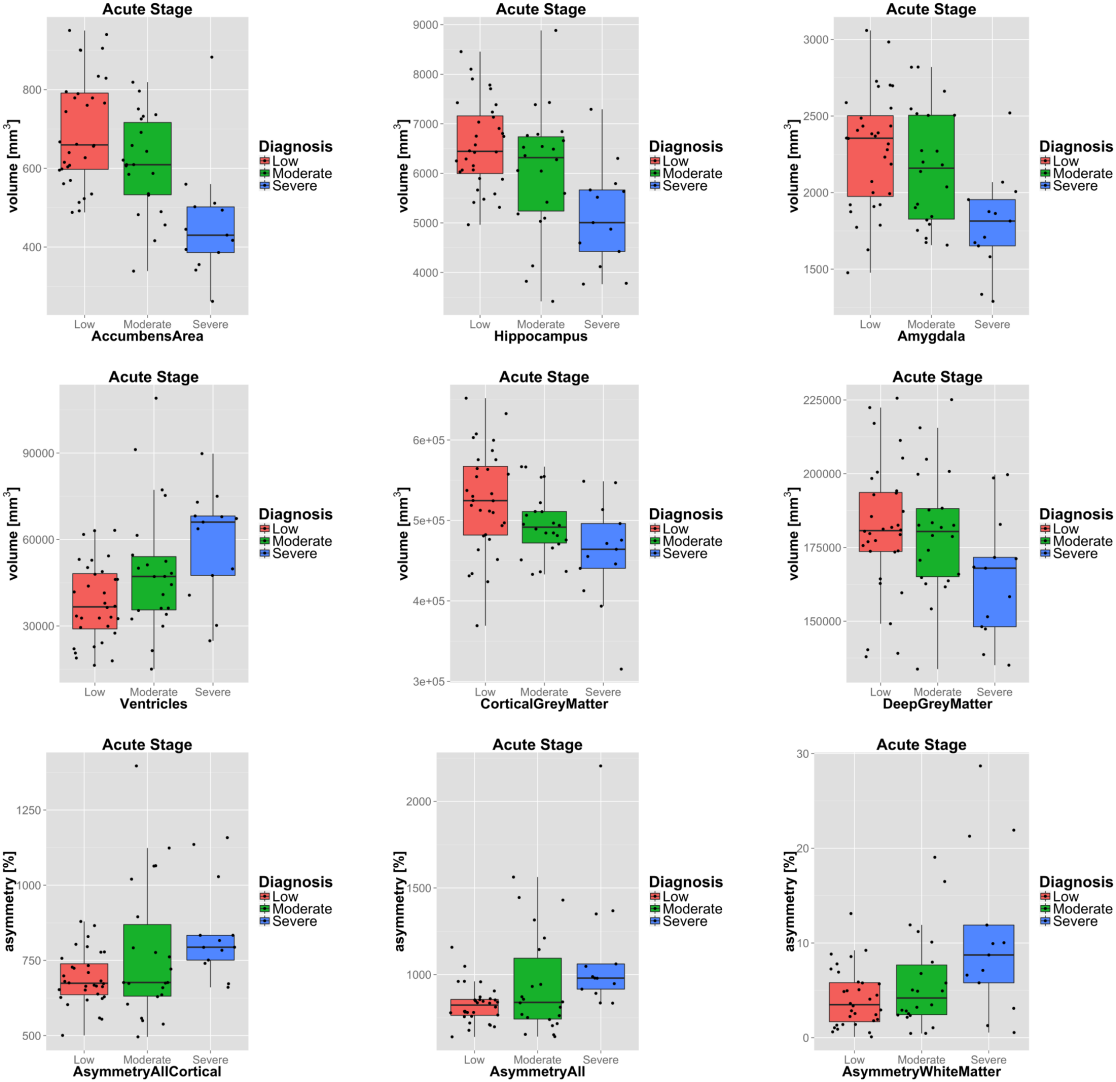
Boxplots of selected imaging features. Boxplots of selected structural volumes (first row), surrogate structures (second row) and asymmetry indices (third row) with respect to the investigated disease outcome groups. Features selected based on their performance in classifying severe disability vs. low disability outcome (c.f. Table 4). Note that absolute asymmetry indices are accumulated for AsymmetryAll and AsymmetryAllCortical and thus greater than 100%.

The distributions of selected structural volumes and AAIs with respect to the three considered outcome categories low, moderate and severe disability are shown in Fig 5. Several subcortical structures were identified that are of particularly small size at the acute injury stage in subjects with severe disability outcome as compared to those patients with a low disability outcome (c.f. Table 4). The four structures with the largest effect size are the accumbens (Cohen’s d = 1.66), hippocampus (d = 1.56), amygdala (d = 1.33) and the thalamus (d = 1.27). These differences are significant after Bonferroni correction for multiple comparisons. Overall, a larger ventricular volume (d = 1.36) and lower cortical GM volume (d = 1.0) was observed among patients with unfavourable outcome as compared to those with a good outcome diagnosis. Asymmetry throughout the whole brain and in particular within the cortex and WM was significantly higher in patients with severe disability outcome than in the low disability outcome group.

**Table 4:** Acute-stage classification results (severe vs. low disability outcome). Classification results in% (6-fold cross-validation, 100 runs) obtained separating TBI patients with a severe disability from patients with low disability outcome based on structural volumes and asymmetry at the acute stage of the injury. Individual structures are sorted by effect size. Significant group differences indicated by “+” (p < 0.05) and “++” (p < 0.001), or “o” if not significant. Bonferroni corrected significance in parentheses. Individual features were classified using LDA, multiple features using RandomForest or SVM.

**Severe disability** (N = 13, Positives^P^) vs. **Low disability** (N = 32, Negatives^N^) (**acute stage**)								
structure	ACC (bACC)	SENS	SPEC	mean (SD) [mm^3^]^P^	mean (SD) [mm^3^]^N^	effect size (d)	p-value	sig. (corr.)
RandomForest (all features)	85 (79)	66	93					
SVM (all features)	87 (84)	76	92					
RandomForest (MALPEM^vol,sym^ only)	85 (79)	65	93					
SVM (MALPEM^vol,sym^ only)	83 (77)	62	92					
RandomForest (clinical only)	82 (78)	69	87					
SVM (clinical only)	82 (76)	62	91					
RandomForest (age and gender only)	69 (60)	39	81					
SVM (age and gender only)	70 (50)	1	99					
MarshallScore	84 (80)	69	91	3.9 (1.4)	1.6 (1.1)	1.907	<0.00001	++ (++)
InjurySeverity	71 (66)	54	78	2.8 (1.0)	2.3 (0.7)	0.724	0.03308	+ (o)
Gender (female = 0, male = 1)	67 (67)	69	66	0.3 (0.5)	0.7 (0.5)	0.723	0.03331	+ (o)
GlasgowComaScale	72 (60)	30	89	12.0 (4.5)	14.1 (2.2)	0.691	0.04153	+ (o)
Age	55 (54)	54	55	67.2 (15.9)	61.1 (10.1)	0.514	0.12518	o (o)
Ventricles	72 (71)	68	74	58766.8 (18808.4)	38131.9 (13556.0)	1.357	0.00017	++ (+)
CorticalGreyMatter	70 (70)	70	71	459934.2 (63395.6)	524318.2 (64687.5)	1.001	0.00398	+ (o)
DeepGreyMatter	74 (74)	74	75	164564.8 (20726.5)	181380.2 (22209.8)	0.771	0.02373	+ (o)
BrainTissue	60 (60)	60	60	1130166.1 (102464.8)	1198466.6 (135087.2)	0.539	0.10886	o (o)
Brain	58 (59)	62	57	1191022.6 (110493.8)	1238799.0 (138060.0)	0.365	0.27348	o (o)
WhiteMatter	50 (46)	37	55	505667.1 (82222.8)	492768.2 (71084.9)	0.173	0.60062	o (o)
AccumbensArea	82 (85)	91	79	460.0 (150.5)	696.3 (138.7)	1.664	<0.00001	++ (++)
Hippocampus	80 (81)	83	79	5136.2 (1030.6)	6572.2 (873.5)	1.561	0.00002	++ (+)
Amygdala	73 (76)	83	68	1795.5 (321.1)	2280.9 (380.7)	1.329	0.00022	++ (+)
LateralVentricle	75 (74)	73	76	51162.3 (17123.4)	32704.8 (12459.3)	1.326	0.00022	++ (+)
InfLatVent	81 (75)	62	89	3131.4 (1360.4)	1913.3 (676.5)	1.324	0.00023	++ (+)
ThalamusProper	73 (74)	76	72	11584.6 (1556.0)	13832.8 (1845.3)	1.271	0.00037	++ (+)
BasalForebrain	74 (75)	77	72	548.7 (232.3)	840.4 (233.9)	1.250	0.00045	++ (+)
CerebellarVermalLobulesI-V	75 (74)	72	76	3471.9 (535.9)	4242.3 (699.1)	1.171	0.00092	++ (+)
3rdVentricle	71 (71)	69	72	2288.6 (814.2)	1552.5 (617.3)	1.086	0.00194	+ (o)
Putamen	76 (76)	77	75	6066.7 (2492.2)	7849.7 (1338.7)	1.025	0.00326	+ (o)
BrainStem	69 (69)	71	68	17417.2 (2275.5)	19651.1 (2739.4)	0.853	0.01291	+ (o)
CerebellumWhiteMatter	73 (72)	69	74	36782.2 (7917.1)	30605.9 (8306.2)	0.753	0.02697	+ (o)
VentralDC	56 (55)	54	56	8247.1 (1062.4)	8961.7 (1114.9)	0.649	0.05477	o (o)
CerebellarVermalLobulesVIII-X	57 (62)	74	51	2659.5 (304.5)	2902.0 (420.4)	0.619	0.06650	o (o)
CerebellumExterior	64 (64)	63	64	94910.8 (12670.3)	101795.8 (13850.8)	0.509	0.12919	o (o)
4thVentricle	60 (55)	45	66	2184.5 (673.2)	1961.2 (526.9)	0.391	0.24153	o (o)
Caudate	53 (47)	35	60	7388.5 (3739.3)	6613.9 (1066.8)	0.356	0.28451	o (o)
Pallidum	52 (51)	48	54	2801.2 (796.4)	2990.8 (504.4)	0.316	0.34230	o (o)
CerebellarVermalLobulesVI-VII	54 (54)	54	54	2076.6 (384.4)	2150.0 (299.1)	0.226	0.49648	o (o)
CSF	47 (45)	42	48	2025.5 (719.9)	2140.6 (538.2)	0.194	0.55923	o (o)
CerebralWhiteMatter	43 (38)	28	49	468884.8 (76690.5)	462162.3 (67838.9)	0.095	0.77302	o (o)
AsymmetryAllCortical^†^	76 (74)	69	79	846.2 (160.7)	690.8 (88.0)	1.374	0.00014	++ (+)
AsymmetryAll^†^	81 (73)	54	91	1108.3 (369.6)	826.6 (107.9)	1.306	0.00027	++ (+)
AsymmetryWhiteMatter	75 (69)	55	82	10.5 (8.5)	4.2 (3.1)	1.220	0.00059	++ (+)
AsymmetryCerebralWhiteMatter	76 (68)	48	88	10.2 (9.5)	3.8 (2.8)	1.150	0.00110	+ (o)
AsymmetryAmygdala	82 (78)	68	87	16.9 (12.5)	7.7 (5.9)	1.119	0.00145	+ (o)
AsymmetryBrain	75 (72)	64	79	4.0 (3.3)	1.6 (1.5)	1.086	0.00194	+ (o)
AsymmetryBrainTissue	81 (73)	54	92	5.2 (5.2)	1.9 (1.7)	1.068	0.00226	+ (o)
AsymmetryCorticalGreyMatter	79 (72)	55	89	4.9 (4.6)	2.0 (2.3)	0.938	0.00663	+ (o)
AsymmetryAllNonCortical^†^	79 (71)	50	91	262.0 (245.1)	135.8 (46.7)	0.932	0.00695	+ (o)
AsymmetryCerebellumWhiteMatter	69 (69)	69	69	19.2 (13.9)	10.5 (7.8)	0.887	0.00997	+ (o)
AsymmetryPutamen	75 (63)	34	92	24.2 (44.3)	4.2 (4.8)	0.844	0.01384	+ (o)
AsymmetryAccumbensArea	66 (62)	53	71	23.4 (18.8)	12.7 (10.2)	0.805	0.01850	+ (o)
AsymmetryCaudate	71 (63)	46	81	17.3 (23.1)	7.2 (6.0)	0.759	0.02585	+ (o)
AsymmetryDeepGreyMatter	72 (66)	52	81	3.9 (4.2)	2.0 (1.9)	0.706	0.03743	+ (o)
AsymmetryThalamusProper	75 (60)	27	94	14.2 (35.4)	2.2 (2.0)	0.642	0.05762	o (o)
AsymmetryVentricles	68 (59)	39	79	27.8 (32.4)	15.4 (13.5)	0.600	0.07507	o (o)
AsymmetryHippocampus	63 (57)	43	71	16.2 (16.2)	9.9 (7.3)	0.595	0.07721	o (o)
AsymmetryPallidum	68 (56)	28	84	15.9 (32.6)	5.5 (5.4)	0.580	0.08469	o (o)
AsymmetryLateralVentricle	66 (57)	38	77	31.5 (35.6)	18.2 (15.6)	0.574	0.08797	o (o)
AsymmetryVentralDC	65 (56)	36	76	9.5 (17.3)	4.2 (2.7)	0.556	0.09791	o (o)
AsymmetryBasalForebrain	63 (55)	37	74	37.3 (40.4)	24.4 (17.1)	0.498	0.13745	o (o)
AsymmetryCerebellumExterior	68 (64)	54	74	4.8 (3.1)	3.5 (3.7)	0.373	0.26322	o (o)
AsymmetryInfLatVent	40 (37)	29	45	21.5 (20.5)	21.6 (17.7)	0.008	0.98119	o (o)

^†^: Sum of the AAIs of the individual structures.

We also identified several significant structural differences when comparing structural volumes at the acute stage with respect to the moderate disability group. The respective classification accuracies, structural volumes and corresponding statistics are summarized in Table 5. For example cerebellar vermal lobules (d = 1.24), accumbens (d = 1.11), amygdala (d = 1.02), thalamus (d = 0.86) and brain stem (d = 0.85) are at the acute disease stage significantly smaller in severe disability outcome patients as compared to patients with moderate disability outcome. We found the most significant differences between moderate and low disability are larger ventricles (d = 0.65), specifically larger inferior lateral ventricles (d = 0.80), and a smaller accumbens (d = 0.65) in the moderate disability group. The group with moderate outcome disability had overall a wider spread (variance) of the measured features and was thus less well separated from both the low and severe outcome groups. Therefore most findings with respect to the moderate disability category were not significant after correcting for multiple comparisons.

**Table 5:** Acute-stage classification results (severe vs. moderate and moderate vs. low disability outcome). Classification results in% (6-fold cross-validation, 100 runs) obtained separating TBI patients with a severe disability from patients with moderate disability outcome based on structural volumes at the acute stage of the injury (top). Classification of patients with moderate and low disability outcome (bottom). Individual structures are sorted by effect size. Significant group differences indicated by + (p < 0.05) and ++ (p < 0.001), or “o” if not significant. Bonferroni corrected significance in parentheses. Individual features were classified using LDA, multiple features using RandomForest or SVM. Results for individual structural asymmetry features are shown in S1 File.

**Severe disability** (N = 13, Positives^P^) vs. **Moderate disability** (N = 22, Negatives^N^) (**cross-sectional analysis, acute stage**)								
structure	ACC (bACC)	SENS	SPEC	mean (SD) [mm^3^]^P^	mean (SD) [mm^3^]^N^	effect size (d)	p-value	sig. (corr.)
RandomForest (all cross-sectional features)	64 (58)	36	81					
SVM (all cross-sectional features)	64 (61)	45	76					
Gender (female = 0, male = 1)	66 (66)	69	64	0.3 (0.5)	0.6 (0.5)	0.673	0.06287	o (o)
Age	64 (63)	55	70	67.2 (15.9)	58.8 (10.0)	0.672	0.06331	o (o)
MarshallScore	57 (58)	62	55	3.9 (1.4)	3.0 (1.9)	0.510	0.15465	o (o)
GlasgowComaScale	43 (38)	20	57	12.0 (4.5)	12.6 (3.9)	0.155	0.66141	o (o)
InjurySeverity	38 (38)	35	40	2.8 (1.0)	2.8 (0.8)	0.083	0.81296	o (o)
DeepGreyMatter	67 (68)	73	63	164564.8 (20726.5)	180076.7 (20887.9)	0.745	0.04082	+ (o)
CorticalGreyMatter	67 (67)	66	68	459934.2 (63395.6)	494829.1 (39983.4)	0.701	0.05339	o (o)
BrainTissue	55 (55)	54	56	1130166.1 (102464.8)	1181045.4 (89378.0)	0.539	0.13272	o (o)
Ventricles	69 (67)	62	73	58766.8 (18808.4)	49592.9 (22303.5)	0.435	0.22267	o (o)
Brain	59 (58)	58	59	1191022.6 (110493.8)	1232890.5 (98415.8)	0.407	0.25346	o (o)
WhiteMatter	35 (34)	30	39	505667.1 (82222.8)	506139.5 (76357.7)	0.006	0.98638	o (o)
CerebellarVermalLobulesVIII-X	77 (78)	84	73	2659.5 (304.5)	3110.6 (394.1)	1.239	0.00121	+ (o)
AccumbensArea	76 (78)	84	71	460.0 (150.5)	609.9 (125.5)	1.110	0.00327	+ (o)
Amygdala	65 (67)	75	59	1795.5 (321.1)	2161.0 (378.9)	1.018	0.00642	+ (o)
ThalamusProper	60 (61)	63	59	11584.6 (1556.0)	13072.5 (1826.3)	0.859	0.01955	+ (o)
BrainStem	61 (62)	67	57	17417.2 (2275.5)	19416.8 (2417.5)	0.845	0.02144	+ (o)
BasalForebrain	63 (66)	77	55	548.7 (232.3)	773.2 (290.7)	0.829	0.02383	+ (o)
Hippocampus	64 (64)	62	66	5136.2 (1030.6)	6033.6 (1261.9)	0.759	0.03743	+ (o)
CerebellarVermalLobulesI-V	63 (64)	68	59	3471.9 (535.9)	3949.6 (704.4)	0.737	0.04283	+ (o)
VentralDC	61 (59)	54	64	8247.1 (1062.4)	8844.7 (880.6)	0.629	0.08149	o (o)
CerebellumExterior	61 (62)	66	57	94910.8 (12670.3)	103457.0 (14443.9)	0.618	0.08647	o (o)
Putamen	73 (74)	76	72	6066.7 (2492.2)	7087.0 (1586.9)	0.519	0.14721	o (o)
Pallidum	54 (54)	54	54	2801.2 (796.4)	3063.0 (442.4)	0.439	0.21786	o (o)
LateralVentricle	69 (68)	62	74	51162.3 (17123.4)	42726.9 (20449.5)	0.437	0.22046	o (o)
CerebellumWhiteMatter	65 (66)	69	63	36782.2 (7917.1)	33401.6 (8837.8)	0.397	0.26456	o (o)
InfLatVent	61 (61)	58	63	3131.4 (1360.4)	2649.1 (1182.9)	0.386	0.27817	o (o)
Caudate	63 (59)	43	74	7388.5 (3739.3)	6400.6 (1557.5)	0.384	0.28063	o (o)
CSF	52 (52)	51	52	2025.5 (719.9)	2194.0 (558.1)	0.271	0.44433	o (o)
3rdVentricle	60 (58)	54	63	2288.6 (814.2)	2076.9 (941.1)	0.236	0.50451	o (o)
4thVentricle	38 (36)	30	42	2184.5 (673.2)	2140.1 (576.0)	0.072	0.83738	o (o)
CerebralWhiteMatter	37 (36)	35	38	468884.8 (76690.5)	472738.0 (70658.1)	0.053	0.88084	o (o)
CerebellarVermalLobulesVI-VII	39 (39)	38	40	2076.6 (384.4)	2096.5 (498.0)	0.043	0.90263	o (o)
**Moderate disability** (N = 22, Positives^P^) vs. **Low disability** (N = 32, Negatives^N^) **(cross-sectional analysis, acute stage)**								
structure	ACC (bACC)	SENS	SPEC	mean (SD) [mm^3^]^P^	mean (SD) [mm^3^]^N^	effect size (d)	p-value	sig. (corr.)
RandomForest (all cross-sectional features)	61 (56)	32	80					
SVM (all cross-sectional features)	68 (64)	43	85					
MarshallScore	72 (68)	45	91	3.0 (1.9)	1.6 (1.1)	0.975	0.00091	++ (+)
InjurySeverity	69 (66)	55	78	2.8 (0.8)	2.3 (0.7)	0.666	0.01981	+ (o)
GlasgowComaScale	65 (60)	32	87	12.6 (3.9)	14.1 (2.2)	0.483	0.08720	o (o)
Age	58 (59)	62	56	58.8 (10.0)	61.1 (10.1)	0.223	0.42386	o (o)
Gender (female = 0, male = 1)	40 (38)	28	48	0.6 (0.5)	0.7 (0.5)	0.041	0.88325	o (o)
Ventricles	61 (61)	58	64	49592.9 (22303.5)	38131.9 (13556.0)	0.650	0.02268	+ (o)
CorticalGreyMatter	66 (67)	71	63	494829.1 (39983.4)	524318.2 (64687.5)	0.526	0.06297	o (o)
WhiteMatter	46 (44)	35	54	506139.5 (76357.7)	492768.2 (71084.9)	0.183	0.51279	o (o)
BrainTissue	49 (49)	49	48	1181045.4 (89378.0)	1198466.6 (135087.2)	0.147	0.59861	o (o)
DeepGreyMatter	42 (41)	38	44	180076.7 (20887.9)	181380.2 (22209.8)	0.060	0.82903	o (o)
Brain	40 (40)	39	40	1232890.5 (98415.8)	1238799.0 (138060.0)	0.048	0.86362	o (o)
InfLatVent	71 (69)	60	78	2649.1 (1182.9)	1913.3 (676.5)	0.804	0.00543	+ (o)
3rdVentricle	61 (61)	56	65	2076.9 (941.1)	1552.5 (617.3)	0.686	0.01660	+ (o)
AccumbensArea	58 (59)	64	53	609.9 (125.5)	696.3 (138.7)	0.647	0.02332	+ (o)
LateralVentricle	62 (61)	57	65	42726.9 (20449.5)	32704.8 (12459.3)	0.620	0.02953	+ (o)
Putamen	57 (56)	51	61	7087.0 (1586.9)	7849.7 (1338.7)	0.528	0.06207	o (o)
Hippocampus	54 (53)	49	58	6033.6 (1261.9)	6572.2 (873.5)	0.514	0.06913	o (o)
CerebellarVermalLobulesVIII-X	65 (65)	66	65	3110.6 (394.1)	2902.0 (420.4)	0.509	0.07185	o (o)
CerebellarVermalLobulesI-V	60 (61)	64	58	3949.6 (704.4)	4242.3 (699.1)	0.417	0.13786	o (o)
ThalamusProper	60 (61)	68	54	13072.5 (1826.3)	13832.8 (1845.3)	0.414	0.14127	o (o)
CerebellumWhiteMatter	56 (54)	45	64	33401.6 (8837.8)	30605.9 (8306.2)	0.328	0.24176	o (o)
4thVentricle	62 (62)	59	65	2140.1 (576.0)	1961.2 (526.9)	0.327	0.24336	o (o)
Amygdala	60 (60)	58	62	2161.0 (378.9)	2280.9 (380.7)	0.315	0.26007	o (o)
BasalForebrain	52 (51)	46	55	773.2 (290.7)	840.4 (233.9)	0.260	0.35230	o (o)
Caudate	51 (51)	50	51	6400.6 (1557.5)	6613.9 (1066.8)	0.166	0.55236	o (o)
CerebralWhiteMatter	43 (41)	33	50	472738.0 (70658.1)	462162.3 (67838.9)	0.153	0.58231	o (o)
Pallidum	49 (48)	41	54	3063.0 (442.4)	2990.8 (504.4)	0.151	0.58912	o (o)
CerebellarVermalLobulesVI-VII	48 (47)	43	52	2096.5 (498.0)	2150.0 (299.1)	0.137	0.62397	o (o)
CerebellumExterior	45 (44)	40	49	103457.0 (14443.9)	101795.8 (13850.8)	0.118	0.67217	o (o)
VentralDC	44 (44)	47	41	8844.7 (880.6)	8961.7 (1114.9)	0.114	0.68245	o (o)
CSF	45 (45)	42	47	2194.0 (558.1)	2140.6 (538.2)	0.098	0.72593	o (o)
BrainStem	44 (44)	47	42	19416.8 (2417.5)	19651.1 (2739.4)	0.090	0.74754	o (o)

We further investigated whether the disability outcome of a patient can be predicted using only features that are available at the acute stage of the injury. Results for the prediction of severe vs. low disability outcome category are summarised in Table 4. The volume of the accumbens provided the best group separation with the highest bACC of 85% (SENS: 91%, SPEC: 79%). Next to the accumbens, several structures were predictive for the disease outcome including hippocampus (bACC: 81%), amygdala (bACC: 76%) and thalamus (bACC: 74%). Available features were combined into multi-feature classifiers. Using a SVM, MALPEM features extracted from imaging yielded similar results (bACC: 77%) to those obtained with clinical information only. Clinical variables and measurements obtained with MALPEM from imaging contained complementary information. Their combination increased classification accuracy to bACC: 84% (SENS: 76%, SPEC: 92%).

The classification results for severe vs. moderate and moderate vs. low disability outcome classification can be found in Table 5. Due to the large variability within the moderate disability outcome category this task is substantially more difficult. For the severe vs. moderate disability classification, we achieved the highest balanced classification accuracy based on volumes of the cerebellar vermal lobules (bACC: 78%) and accumbens (bACC: 78%). For the moderate vs. low disability classification, we observed the highest accuracy for the volume feature of the inferior lateral ventricle (bACC: 69%).

### 4.2 Longitudinal analysis

**Fig 6:**
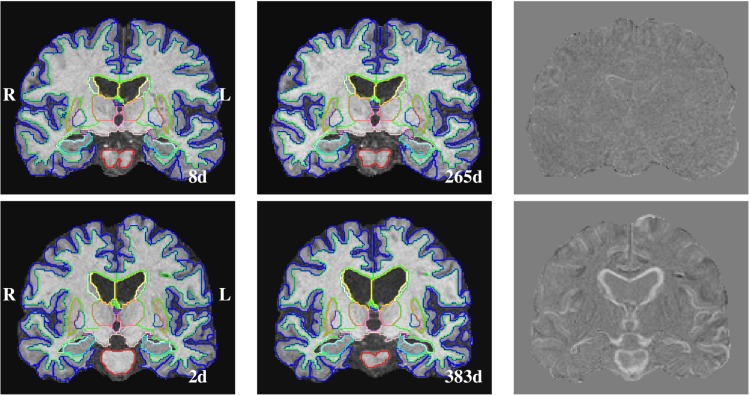
Example longitudinal segmentation results. Segmentation results shown of images acquired at the acute and chronic stage of a TBI. Top: TBI061, male, 69 years of age, GCS: 14, GOSe: 8, MCS: 1, fall accident, acute/chronic image acquired 8/265 days after injury. Bottom: TBI142, male, 51 years of age, GCS: 3, GOSe: 6, MCS: 5, transport accident, acute/chronic image acquired 2/383 days after injury, diffuse axonal injury. The difference image of subject TBI142 illustrates the clear ventricular enlargement (measured: 47%), hippocampal atrophy (-6.2%) and reduction of brain stem volume (-13%). The colour scheme is described in S1 File.

Further a longitudinal analysis was performed to investigate the volume change of individual ROIs between the acute stage of the injury and the follow-up visit in the chronic phase of the disease. Example longitudinal segmentation results obtained on two subjects with low and moderate disability outcome are shown in Fig 6.

**Fig 7:**
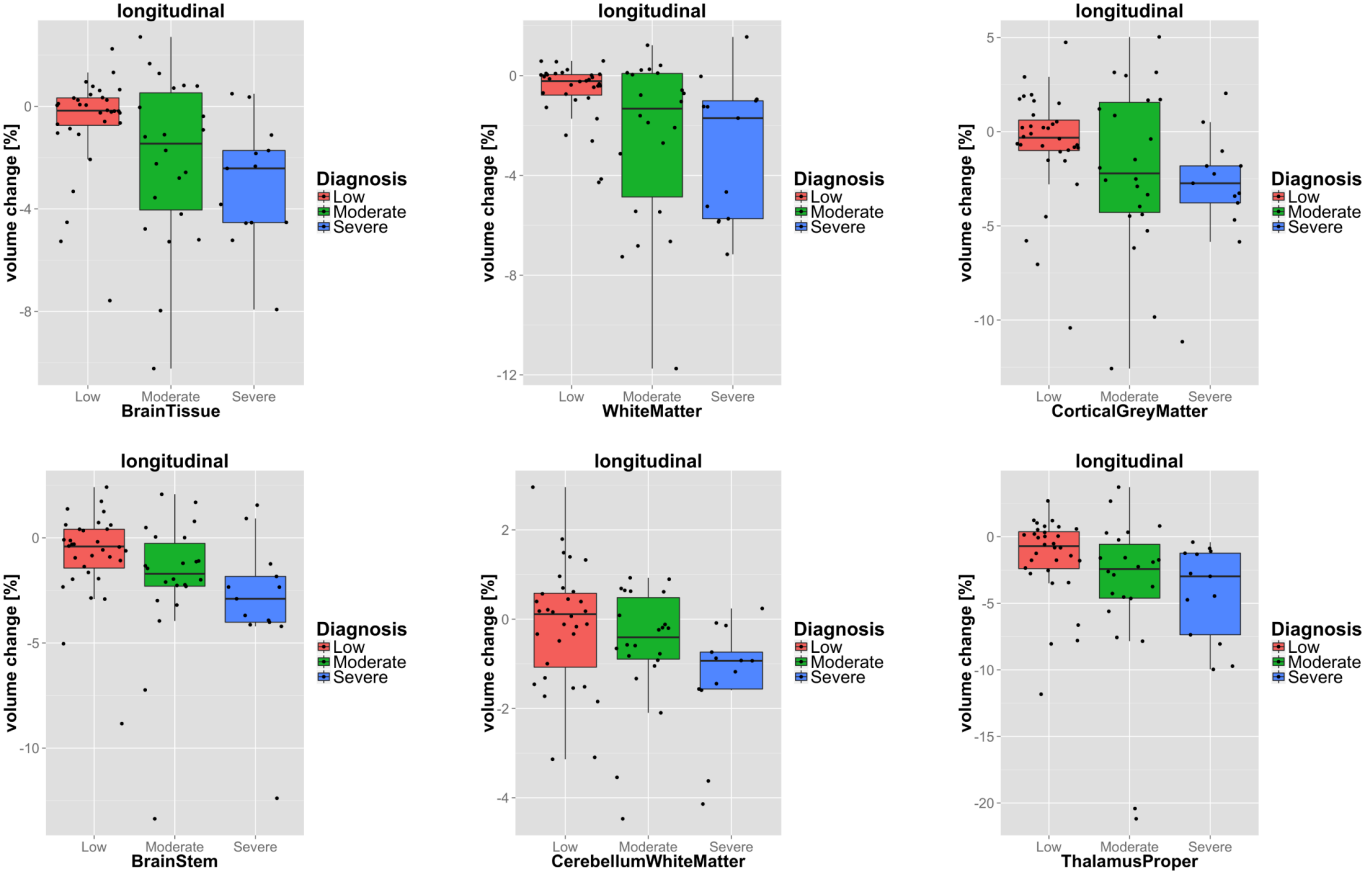
Boxplots of change rates. Change rates of selected ROIs with respect to the investigated disease outcome groups. Features selected based on their performance in classifying severe disability vs. low disability outcome (c.f. Table 6).

The relation of structural atrophy rates and patient groups of distinct disease outcome was investigated. Specifically, classification accuracies, effect sizes and p-values were calculated to quantify group separation. The distribution of the volume change of six selected structures is shown in Fig 7 for the three considered outcome groups.

The quantitative results for the comparison of severe and low disability outcome are summarised in Table 6. Substantial ventricular expansion (11.6%) was observed in patients with poor disease outcome. In contrast brain tissue and in particular cerebral WM (-3.1%), brain stem (-3.1%) and thalamus (-4.2%) showed increased atrophy.

**Table 6:** Longitudinal classification results (severe vs. low disability outcome). Classification results in% (6-fold cross-validation, 100 runs) obtained separating TBI patients with a severe disability from patients with low disability outcome based on structural volume changes between the acute and chronic disease stage. Significant group differences indicated by + (p < 0.05) and ++ (p < 0.001), or “o” if not significant. Bonferroni corrected significance in parentheses. Individual features were classified using LDA, multiple features using RandomForest or SVM.

**Severe disability** (N = 13, Positives^P^) vs. **Low disability** (N = 32, Negatives^N^) (**longitudinal analysis**, bl→follow-up)								
structure	ACC (bACC)	SENS	SPEC	mean (SD) [%]^P^	mean (SD) [%]^N^	effect size (d)	p-value	sig. (corr.)
RandomForest (all features)	77 (69)	52	86					
SVM (all features)	75 (64)	36	91					
Gender (female = 0, male = 1)	67 (67)	69	66	0.3 (0.5)	0.7 (0.5)	0.723	0.03331	+ (o)
Age	55 (55)	54	55	67.2 (15.9)	61.1 (10.1)	0.514	0.12518	o (o)
WhiteMatter	75 (67)	47	87	-3.0 (2.8)	-0.6 (1.2)	1.323	0.00023	++ (+)
BrainTissue	79 (75)	66	84	-3.0 (2.4)	-0.6 (2.0)	1.130	0.00133	+ (+)
Brain	80 (77)	70	84	-2.0 (1.7)	-0.4 (1.6)	1.020	0.00339	+ (o)
DeepGreyMatter	70 (69)	64	73	-2.7 (2.2)	-0.5 (2.5)	0.896	0.00929	+ (o)
CorticalGreyMatter	78 (75)	66	84	-3.0 (3.2)	-0.7 (2.9)	0.780	0.02223	+ (o)
Ventricles	68 (63)	50	76	11.6 (12.0)	4.2 (9.7)	0.711	0.03627	+ (o)
CerebralWhiteMatter	75 (67)	47	87	-3.1 (3.0)	-0.7 (1.2)	1.293	0.00030	++ (+)
CerebellumExterior	70 (67)	60	74	-3.1 (3.1)	-0.3 (2.9)	0.933	0.00691	+ (o)
BrainStem	78 (76)	70	81	-3.1 (3.3)	-0.8 (2.1)	0.922	0.00758	+ (o)
CerebellumWhiteMatter	72 (72)	73	72	-1.3 (1.3)	-0.1 (1.3)	0.884	0.01019	+ (o)
ThalamusProper	72 (66)	52	80	-4.2 (3.5)	-1.6 (3.1)	0.824	0.01605	+ (o)
LateralVentricle	69 (63)	51	76	12.8 (12.0)	4.7 (10.7)	0.725	0.03289	+ (o)
Amygdala	57 (47)	26	69	-2.6 (6.7)	0.0 (2.5)	0.622	0.06518	o (o)
Hippocampus	73 (65)	46	83	-4.8 (10.6)	-1.1 (2.9)	0.614	0.06895	o (o)
CerebellarVermalLobulesVI-VII	68 (65)	58	72	-2.1 (3.9)	-0.1 (3.1)	0.613	0.06917	o (o)
CSF	66 (61)	49	72	6.2 (6.5)	3.2 (6.0)	0.500	0.13556	o (o)
Putamen	58 (51)	34	67	-0.1 (0.8)	0.1 (0.4)	0.497	0.13840	o (o)
VentralDC	76 (74)	68	79	-2.5 (1.8)	-1.2 (3.0)	0.480	0.15191	o (o)
4thVentricle	61 (61)	61	61	0.3 (7.5)	-2.2 (4.5)	0.468	0.16234	o (o)
InfLatVent	69 (64)	52	76	7.7 (19.6)	3.0 (8.0)	0.380	0.25411	o (o)
CerebellarVermalLobulesI-V	59 (57)	52	62	-0.9 (1.6)	-0.5 (1.0)	0.355	0.28602	o (o)
CerebellarVermalLobulesVIII-X	65 (64)	62	66	-0.3 (1.9)	0.4 (2.3)	0.331	0.32032	o (o)
3rdVentricle	56 (53)	45	60	5.2 (8.4)	2.4 (9.2)	0.316	0.34251	o (o)
Caudate	39 (37)	30	43	0.1 (12.6)	-1.1 (6.6)	0.132	0.69072	o (o)
AccumbensArea	49 (47)	43	52	-0.2 (3.0)	-0.5 (1.7)	0.118	0.72056	o (o)
BasalForebrain	44 (41)	34	48	1.2 (16.6)	2.7 (14.6)	0.096	0.77201	o (o)
Pallidum	56 (44)	15	72	0.2 (0.8)	0.2 (1.1)	0.023	0.94465	o (o)

The clearest group separation was calculated for atrophy of the cerebral WM (d = 1.29, p ≈ 10^−3^). Volumetric change of individual ROIs, such as the thalamus, brain stem and cerebellum WM were significantly different (d > 0.8, p < 0.05) between patients with low and severe outcome disability. Significant group differences were found for cortical GM atrophy and ventricular enlargement with effect sizes above 0.7. Cerebral WM is, however, the only individual structure which remained statistically different between the groups after correcting for multiple comparisons.

The most discriminative structure in terms of accuracy was atrophy of the whole brain (bACC: 77%, SENS: 70%, SPEC: 84%) and the brain stem (bACC: 76%, SENS: 70%, SPEC: 81%). A combination of all measured atrophy rates in a multi-feature classifiers did not further improve classification results, e.g. using a RF classifier resulted in bACC: 69%.

Quantitative results for severe vs. moderate and moderate vs. low outcome disability can be found in Table 7. We found a significant reduction in white matter volume (d = 0.82) in patients with moderate disability (-2.5%) as compared to patients with low outcome disability (-0.6%). Patients with severe outcome disability showed similar ventricular atrophy as patients moderate outcome disability (11.6%). Further, even though not significant (p ≈ 0.08), we observed a substantially higher reduction in deep grey matter volume in patients with severe outcome disability (-2.7% vs. -1.2%).

**Table 7:** Longitudinal classification results (severe vs. moderate and moderate vs. low disability outcome). Classification results in% (6-fold cross-validation, 100 runs) obtained separating TBI patients with a severe disability from patients with moderate disability outcome based on structural volume changes between the acute and chronic disease stage (top). Classification of patients with moderate disability and low disability outcome (bottom). The individual structures are sorted by effect size. Significant group differences indicated by + (p < 0.05) and ++ (p < 0.001), or “o” if not significant. Bonferroni corrected significance in parentheses. Individual features were classified using LDA, multiple features using RandomForest or SVM.

**Severe disability** (N = 13, Positives^P^) vs. **Moderate disability** (N = 22, Negatives^N^) **(longitudinal analysis, bl→follow-up)**								
structure	ACC (bACC)	SENS	SPEC	mean (SD) [%]^P^	mean (SD) [%]^N^	effect size (d)	p-value	sig. (corr.)
RandomForest (all features)	59 (53)	29	76					
SVM (all features)	56 (50)	25	75					
Gender (female = 0, male = 1)	66 (66)	69	64	0.3 (0.5)	0.6 (0.5)	0.673	0.06287	o (o)
Age	65 (63)	55	70	67.2 (15.9)	58.8 (10.0)	0.672	0.06331	o (o)
DeepGreyMatter	62 (59)	48	70	-2.7 (2.2)	-1.2 (2.3)	0.639	0.07664	o (o)
Brain	61 (62)	65	59	-2.0 (1.7)	-1.3 (2.3)	0.352	0.32098	o (o)
BrainTissue	56 (54)	48	61	-3.0 (2.4)	-2.1 (3.2)	0.310	0.38205	o (o)
CorticalGreyMatter	51 (51)	51	52	-3.0 (3.2)	-1.9 (4.3)	0.279	0.43074	o (o)
WhiteMatter	49 (46)	37	56	-3.0 (2.8)	-2.5 (3.3)	0.154	0.66288	o (o)
Ventricles	37 (36)	35	37	11.6 (12.0)	11.6 (17.8)	0.001	0.99774	o (o)
CerebellumExterior	69 (66)	57	75	-3.1 (3.1)	-0.6 (2.4)	0.933	0.01173	+ (o)
CerebellarVermalLobulesVI-VII	60 (60)	61	59	-2.1 (3.9)	0.4 (3.8)	0.668	0.06477	o (o)
CerebellumWhiteMatter	67 (64)	52	75	-1.3 (1.3)	-0.6 (1.4)	0.535	0.13539	o (o)
Caudate	50 (51)	53	48	0.1 (12.6)	-4.7 (12.3)	0.385	0.27928	o (o)
BrainStem	65 (62)	52	73	-3.1 (3.3)	-2.0 (3.2)	0.332	0.34973	o (o)
AccumbensArea	47 (55)	89	22	-0.2 (3.0)	-3.6 (13.1)	0.316	0.37305	o (o)
Hippocampus	58 (53)	36	70	-4.8 (10.6)	-2.2 (6.7)	0.313	0.37795	o (o)
VentralDC	68 (67)	62	72	-2.5 (1.8)	-1.4 (4.3)	0.294	0.40675	o (o)
Pallidum	37 (46)	78	13	0.2 (0.8)	0.8 (3.2)	0.233	0.50989	o (o)
BasalForebrain	50 (50)	47	52	1.2 (16.6)	5.2 (17.1)	0.233	0.51037	o (o)
CerebellarVermalLobulesI-V	42 (42)	44	40	-0.9 (1.6)	-1.3 (2.1)	0.201	0.56983	o (o)
CerebellarVermalLobulesVIII-X	43 (43)	46	41	-0.3 (1.9)	-0.9 (3.9)	0.187	0.59611	o (o)
Amygdala	44 (40)	21	58	-2.6 (6.7)	-1.6 (5.1)	0.168	0.63403	o (o)
CSF	54 (51)	40	62	6.2 (6.5)	4.7 (10.8)	0.160	0.65057	o (o)
4thVentricle	45 (47)	54	41	0.3 (7.5)	2.3 (15.1)	0.153	0.66533	o (o)
CerebralWhiteMatter	47 (45)	35	54	-3.1 (3.0)	-2.7 (3.6)	0.138	0.69533	o (o)
3rdVentricle	45 (46)	52	41	5.2 (8.4)	6.8 (17.0)	0.112	0.75025	o (o)
Putamen	37 (43)	64	22	-0.1 (0.8)	-0.3 (2.2)	0.071	0.84134	o (o)
ThalamusProper	41 (39)	33	45	-4.2 (3.5)	-3.9 (6.2)	0.053	0.88026	o (o)
LateralVentricle	37 (37)	34	40	12.8 (12.0)	12.5 (18.8)	0.018	0.95937	o (o)
InfLatVent	38 (39)	41	37	7.7 (19.6)	7.8 (24.3)	0.006	0.98701	o (o)
**Moderate disability** (N = 22, Positives^P^) vs. **Low disability** (N = 32, Negatives^N^) **(longitudinal analysis, bl→follow-up)**								
structure	ACC (bACC)	SENS	SPEC	mean (SD) [%]^P^	mean (SD) [%]^N^	effect size (d)	p-value	sig. (corr.)
RandomForest (all features)	62 (59)	47	72					
SVM (all features)	65 (58)	21	94					
Age	59 (59)	62	56	58.8 (10.0)	61.1 (10.1)	0.223	0.42386	o (o)
Gender (female = 0, male = 1)	39 (38)	30	46	0.6 (0.5)	0.7 (0.5)	0.041	0.88325	o (o)
WhiteMatter	69 (66)	47	85	-2.5 (3.3)	-0.6 (1.2)	0.820	0.00460	+ (o)
BrainTissue	70 (67)	51	84	-2.1 (3.2)	-0.6 (2.0)	0.570	0.04449	+ (o)
Ventricles	62 (60)	44	76	11.6 (17.8)	4.2 (9.7)	0.545	0.05460	o (o)
Brain	72 (69)	55	83	-1.3 (2.3)	-0.4 (1.6)	0.461	0.10190	o (o)
CorticalGreyMatter	70 (68)	58	78	-1.9 (4.3)	-0.7 (2.9)	0.346	0.21716	o (o)
DeepGreyMatter	59 (57)	48	66	-1.2 (2.3)	-0.5 (2.5)	0.300	0.28432	o (o)
CerebralWhiteMatter	69 (66)	47	85	-2.7 (3.6)	-0.7 (1.2)	0.817	0.00474	+ (o)
LateralVentricle	59 (56)	36	75	12.5 (18.8)	4.7 (10.7)	0.531	0.06072	o (o)
CerebellarVermalLobulesI-V	61 (59)	48	69	-1.3 (2.1)	-0.5 (1.0)	0.527	0.06274	o (o)
ThalamusProper	65 (62)	45	78	-3.9 (6.2)	-1.6 (3.1)	0.515	0.06844	o (o)
BrainStem	66 (64)	54	74	-2.0 (3.2)	-0.8 (2.1)	0.470	0.09576	o (o)
4thVentricle	60 (58)	50	67	2.3 (15.1)	-2.2 (4.5)	0.444	0.11507	o (o)
CerebellarVermalLobulesVIII-X	64 (61)	48	74	-0.9 (3.9)	0.4 (2.3)	0.441	0.11719	o (o)
Amygdala	58 (57)	53	61	-1.6 (5.1)	-0.0 (2.5)	0.424	0.13221	o (o)
Caudate	59 (57)	43	70	-4.7 (12.3)	-1.1 (6.6)	0.389	0.16652	o (o)
AccumbensArea	59 (53)	20	87	-3.6 (13.1)	-0.5 (1.7)	0.367	0.19060	o (o)
3rdVentricle	60 (58)	47	68	6.8 (17.0)	2.4 (9.2)	0.345	0.21816	o (o)
CerebellumWhiteMatter	59 (58)	50	66	-0.6 (1.4)	-0.1 (1.3)	0.341	0.22407	o (o)
InfLatVent	58 (53)	31	76	7.8 (24.3)	3.0 (8.0)	0.291	0.29794	o (o)
Pallidum	55 (49)	14	83	0.8 (3.2)	0.2 (1.1)	0.287	0.30512	o (o)
Putamen	60 (57)	38	75	-0.3 (2.2)	0.1 (0.4)	0.271	0.33165	o (o)
Hippocampus	56 (53)	36	70	-2.2 (6.7)	-1.1 (2.9)	0.237	0.39550	o (o)
CSF	51 (48)	32	64	4.7 (10.8)	3.2 (6.0)	0.188	0.49978	o (o)
BasalForebrain	51 (50)	47	53	5.2 (17.1)	2.7 (14.6)	0.159	0.56781	o (o)
CerebellarVermalLobulesVI-VII	44 (43)	40	47	0.4 (3.8)	-0.1 (3.1)	0.151	0.58850	o (o)
CerebellumExterior	47 (46)	39	52	-0.6 (2.4)	-0.3 (2.9)	0.103	0.71168	o (o)
VentralDC	49 (47)	36	57	-1.4 (4.3)	-1.2 (3.0)	0.066	0.81394	o (o)

## 5 Discussion

**Fig 8:**
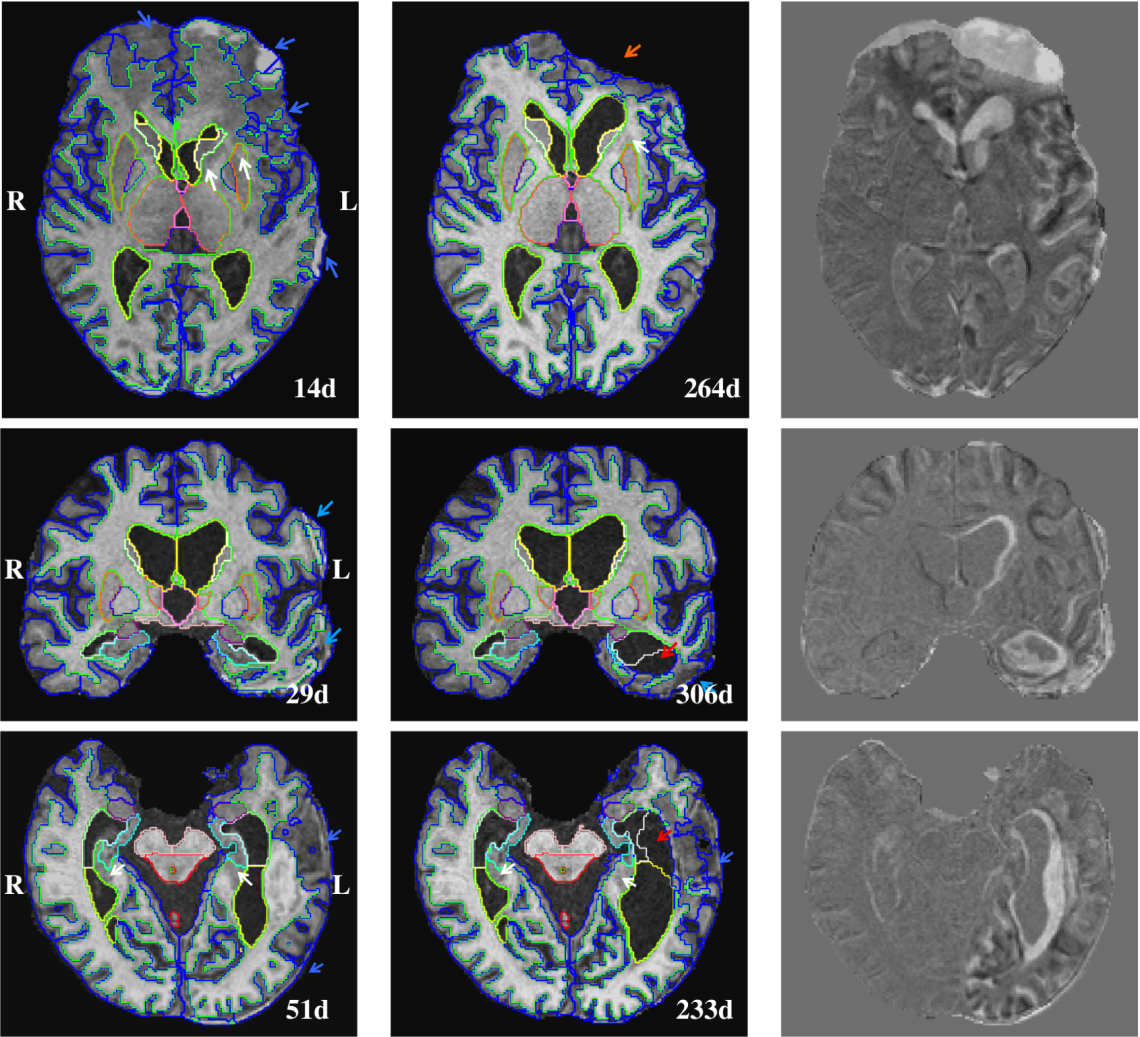
Example longitudinal segmentation results. Images acquired at the acute and chronic stage of a TBI. Top: TBI150, male, 68 years of age, GCS: 15, GOSe: 5, MCS: 5, unclear accident, acute/chronic image acquired 14/264 days after injury, craniotomy and evacuation of an acute SDH, intracranial haematoma induced brain destruction. These are incomplete reproductions of the radiological reports. Middle: TBI047, male, 62 years of age, GCS: 13, GOSe: 3, MCS: 5, fall accident, acute/chronic image acquired 29/306 days after injury, craniotomy and evacuation of an acute subdural haemorrhage, broad gliosis on left temporal lobe. Bottom: TBI114, male, 69 years of age, GCS: 14, GOSe: 5, MCS: 5, cycling accident, acute/chronic image acquired 51/233 days after injury, no craniotomy, traumatic subarachnoid haemorrhage, intracranial haematoma with surrounding oedema in left temporal lobe, central and cortical atrophy. The colour scheme is described in S1 File.

The potential presence of pathologies such as haemorrhage lesions, contusions or a substantial midline shift pose particular challenges for the analysis of brain MRI in TBI. Images of three example patients with a high degree of injury are visualised with overlaid segmentation results in Fig 8. Clinical information and a brief description of the images is provided in the figure caption.

These examples show clear segmentation inaccuracies observed for example for the hippocampus, putamen or the caudate (white arrows in Fig 8). The inclusion of haemorrhage lesions in both WM and cortical GM ROIs (blue arrows in Fig 8) is a limitation of the proposed segmentation framework. This is expected as atlas-based approaches are restricted to the labelling of structures that are represented in the reference atlases. Assuming that explicit segmentations of pathologies (e.g. lesions) are available (e.g. via dedicated lesion segmentation [69]), this allows to determine the location of lesions relative to anatomical structure. However, in the current approach the presence of pathology can lead to a substantial bias as it can potentially increase a structure’s volume (e.g. cortical GM, blue arrows). The inclusion or exclusion of pathology in the brain mask (orange arrow) can have an even more severe effect. It can lead, as shown in the top row of Fig 8, to an overestimation of the reduction of brain tissue or WM volume.

However, with MALPEM we investigated volumetric and atrophy features of 27 anatomical structures of which most are not affected by pathology. We emphasize that most brain regions are reasonably segmented and therefore have value when analysing structural changes throughout the whole brain. A main focus of the conducted study is to perform a population analysis where we compare patient groups of different disease outcome severity. Despite the limitation that individual volume or atrophy features might be biased due to segmentation inaccuracies caused by pathology the conducted, thorough statistical analysis confirmed the significance of many findings. The explicit segmentation of pathology or structures that are not available in the atlases is highly desirable in this context and the focus of our current research [69–71]. In future we aim to combine frameworks for the segmentation of anatomical brain structure (MALPEM) [53] and TBI related pathology (DeepMedic) [69, 71]. DeepMedic was trained on a dataset acquired in Addenbrooke’s Hospital, Cambridge, UK from patients with moderate to severe TBI on a Siemens 3T TIM Trio scanner. Before applying DeepMedic to a dataset with different characteristics, that result from, e.g., variations in injury severity, scanner model or acquisition parameters further research is needed to address the challenging problem of domain shift [71].

Another limitation is related to the refinement of spatial priors [53]. The refinement of priors compensates inaccuracies in the atlas alignment based on the intensity profiles within the MR image. However, priors between labels with similar intensity profile are not corrected. In extreme cases this can lead to mislabeling of these structures (e.g. CSF ↔ background). An example can be observed in the segmentation results of TBI047 (middle row in Fig 8) or TBI114 (bottom row) where parts of the inferior lateral ventricle are incorrectly labelled as background (c.f. red arrow). It generally depends on the specific question that is investigated whether a non-healthy area should be characterised as pathological lesion, segmented as the tissue that it used to be before the injury or masked out/disregarded completely. We are not aware of approaches that reliably show whether a lesion during the acute stage represents “lost”/non-functional tissue or tissue that is still functional and/or reversible. For instance, some non-healthy tissue in the acute stage may still be partially fulfilling its functions. On the other hand, lesions that are clearly visible on acute MRI may disappear later on. However, whether this represents functional recovery or not is not known since axonal pathology may remain despite visual normality. In our approach, MALPEM tends to classify the dead-core or other pathologies appearing dark on T1-weighted MRI as background and generally assigns the most likely tissue class for possibly functional areas.

In this study, the segmentation quality could not be evaluated quantitatively. Manual reference segmentations of the images were not available and a quantitative quality assessment through experts is prohibitive. However, a quantitative assessment of segmentation accuracy obtained with the employed methods was done in earlier work on different datasets [53, 58]. Further to that, a non-clinician visually reviewed the segmentations calculated in this study to rule out gross failures. C.f. Figs 4, 6 and 8 for included subjects with segmentations considered successful, however, affected by pathology.

We further assessed the value of the calculated segmentations indirectly. Instead of quantifying the actual segmentation quality, the disease-relevance of extracted measurements in the context of classification was analysed. Specifically, several classification experiments were conducted to investigate whether measures of structural volume, asymmetry and volume change are meaningful features in patients with TBI. In this setup both the assessment of segmentation accuracy and the validation of measured features as predictors of disease outcome are coupled a priori. With the outlined limitations regarding segmentation quality in the presence of pathology in mind, the goal of the performed analysis was to show the potential of derived quantitative measures as valid biomarkers that are predictive for the outcome of a head trauma.

Quantified features extracted from acute MR imaging allowed a specific prediction whether a patient will have a negative outcome diagnosis (SPEC: 93%) when classified against the low disability outcome group. In this experiment a high sensitivity was observed using volumes of structural ROIs (e.g. SENS accumbens: 91%, SENS hippocampus: 83%) and high specificity using symmetry-based features (e.g. SPEC AAI of all structures: 91%). This suggests that brain symmetry is only a “necessary” criterion for a favourable disease outcome while asymmetry is a “sufficient” criterion for unfavourable outcome. This is in agreement with findings presented in Ledig et al. [53] on another TBI dataset. However, it must be noted that there is a trade-off between SENS and SPEC, which should be further investigated using for example ROC curves.

In Strangman et al. [33] the authors reported that structural volumes correlate with the potential of patients to recover within a memory rehabilitation program. In that study, structural volumes were extracted from 50 TBI patients several years after the injury in the chronic stage. The authors raise the question whether structural volumes extracted at the acute stage have similar potential. The results of the presented study are encouraging: structural volumes (e.g. hippocampus, thalamus) were identified that are predictive for the outcome of the disease. These findings agree with those presented in Strangman et al. [33]. The influence of brain capacity/reserve on the ability to recover from a TBI needs to be further investigated.

The conducted longitudinal analysis showed increased atrophy after sustaining TBI in the WM, brainstem and thalamus which was also shown in other studies [16, 44]. In contrast to Warner et al. [16], significant changes for the cerebellum but not for the amygdala were found. However, when comparing to other studies the substantial heterogeneity of TBI studies must be considered: In comparison to the study presented in here, Warner et al. [16] analysed 25 patients with DAI and 22 age-matched controls where patients had a lower GCS of 6.2±4.5 (mean±SD) and were much younger (26.8±11.3 years).

In the presented analyses, the significance of many findings could not be confirmed after correcting for multiple comparisons. However, the performed Bonferroni correction is very conservative and the calculated effect sizes indicate that significance levels could be increased on a larger cohort. In summary, the experiments confirm that the developed algorithms can be valuable when automatically analysing cohorts with images covering a wide range of significantly altered brain anatomy.

A simple approach was chosen to account for the uneven age distribution between patients of the investigated outcome categories. This resulted in a substantial reduction of the number of study subjects (N = 114→N = 67). Even though no subjects were excluded from the severe disability outcome group (N = 13), this is a limitation of the conducted experiments as the number of subjects (or samples) per group is small. In the future the applicability of more sophisticated methods to adjust for selection bias, such as inverse-probability weighting [72], should be explored.

In addition to the performed age matching, experiments were carried out to employ regression models trained on healthy control subjects from the Alzheimer’s Disease Neuroimaging Initiative (ADNI) cohort to account for differences in age, gender and brain size. Preliminary results suggested that correction for brain size is not beneficial. One possible explanation is that the calculated brain size estimate may be distorted by TBI-related brain pathology, such as lesions or contusions. Correcting for either age and gender did not substantially alter the results presented in Table 4. Correcting for both age *and* gender using a multi-variate regression approach reduced the discriminative value of the investigated structures. It should be noted that the ADNI subjects are overall older than the TBI subjects so that the trained model needs to be extrapolated in order to be applicable to the TBI dataset. One hypothesis is therefore that the regression model trained on ADNI data is not straightforwardly applicable, due to confounding cohort differences in patient age but also acquisition protocol. These effects require a deeper analysis so that no explicit correction for age or gender was performed.

No clear benefit was observed of combining all available measurements within multi-feature classifiers except when predicting severe and low disability outcome at baseline based on structural volumes. Classifiers were not explicitly tuned. Given the size of the study cohort and the large number of features a lack of generalisation of the classifiers/overfitting might be a problem that should be further investigated in the future.

In clinical practice, both outcome prediction of acute TBI and assessment of TBI-related sequels are still major challenges. Therefore, we feel that the presented results have clinical value as a significant step towards creating more reliable predictive models and providing tools to assess TBI-related outcome. The main merits of our study are in developing and employing a reproducible method for automatic volume measurement that takes into account the very complex pathological anatomy of TBI, and its application in a well-characterised patient population. A shortcoming in our study is the heterogeneous age distribution in relation to the severity. Future studies are required to increase certainty in detecting the TBI-related alterations irrespective of age and TBI severity.

## 6 Conclusion

In this work we analysed 67 subjects from a recently acquired cohort of mild to severe TBI patients. The conducted analyses demonstrate that the employed methodology [53, 58] has the potential to extract meaningful biomarkers from MR brain images such as the volume or volumetric change of individual ROIs. It was confirmed that automatically quantified imaging information can add predictive value when performing an outcome prognosis at the acute stage of the injury. Structural volumes, measured from acute MR images, of the accumbens, hippocampus, amygdala and thalamus were related to the disease outcome. Both white matter and brain atrophy was increased in patients with unfavourable outcome diagnosis. Overall, the employed methodology has, within the discussed limitations, potential to support automated brain morphometry in patients with TBI. An essential prerequisite for a more accurate analysis of abnormal brain images is the integration of recently proposed methodology that is able to explicitly segment disease-related pathology.

## Supporting information

S1 File Supplementary material. S1 Table 1. Non-cortical structures defined in NMM brain atlas. • S1 Table 2. Cortical structures defined in NMM brain atlas. • S1 Table 3. Marshall Classification System. • S1 Table 4. Glasgow Outcome Scale. • S1 Table 5. Acute-stage classification results (severe vs. moderate disability outcome). • S1 Table 6. Acute-stage classification results (moderate vs. low disability outcome) (PDF).
(PDF)
