# Supplementary material for: Regional brain morphometry in patients with traumatic brain injury based on acute- and chronic-phase magnetic resonance imaging
Source: PLoS One. 2017 Nov 28;12(11):e0188152. doi: 10.1371/journal.pone.0188152 (PMC5705131; doi:10.1371/journal.pone.0188152)
Supplement: S1 File — S1 Table 1. Non-cortical structures defined in NMM brain atlas. • S1 Table 2. Cortical structures defined in NMM brain atlas. • S1 Table 3. Marshall Classification System. • S1 Table 4. Glasgow Outcome Scale. • S1 Table 5. Acute-stage classification results (severe vs. moderate disability outcome). • S1 Table 6. Acute-stage classification results (moderate vs. low disability outcome) (PDF). (PDF) [file pone.0188152.s001.pdf]

Supporting Information - Regional brain morphometry in patients with traumatic brain injury based on acute- and chronic-phase magnetic resonance imaging

Christian Ledig[1,*], Konstantinos Kamnitsas[1], Juha Koikkalainen[2,3], Jussi P. Posti[4,5,6], Riikka S. K. Takala[7], Ari Katila[7], Janek Frantzén[4,5,6], Henna Ala-Seppälä[4], Anna Kyllönen[4], Henna-Riikka Maanpää[4], Jussi Tallus[4], Jyrki Lötjönen[2,3], Ben Glocker[1], Olli Tenovuo[4,5], Daniel Rueckert[1]

1 Imperial College London, Department of Computing, London, United Kingdom
2 Combinostics, Tampere, Finland
3 VTT Technical Research Centre of Finland, Tampere, Finland
4 Department of Clinical Medicine, University of Turku, Turku, Finland
5 Division of Clinical Neurosciences, Turku Brain Injury Centre, Turku University Hospital, Turku, Finland
6 Division of Clinical Neurosciences, Department of Neurosurgery, Turku University Hospital, Turku, Finland
7 Perioperative Services, Intensive Care Medicine and Pain Management, Turku University Hospital and University of Turku, Turku, Finland

* ledig.christian@gmail.com, work was done while Christian Ledig was employed at Imperial College London, 180 Queen's Gate, London, SW7 2AZ, United Kingdom

Supplementary Material

Table 1. Non-cortical structures defined in NMM brain atlas. Structure names and how each individual structure was also considered as part of larger surrogate structures in this study. Original ROI IDs were remapped to be consecutive. Colours correspond to segmentation contours in the main manuscript.

**non-cortical** structure	colour of contour	abbreviation	ID (right/left)	Part of surrogate structure
3rd ventricle		3rdVent	1	Ventricles, Brain
4th Ventricle		4thVent	2	Ventricles, Brain
Accumbens Area		AccA	3/4	DeepGreyMatter, BrainTissue, Brain
Amygdala		Am	5/6	DeepGreyMatter, BrainTissue, Brain
Brain Stem		BS	7	DeepGreyMatter, BrainTissue, Brain
Caudate		Cau	8/9	DeepGreyMatter, BrainTissue, Brain
Cerebellum Exterior		CblmExt	10/11	DeepGreyMatter, BrainTissue, Brain
Cerebellum White Matter		CblmWM	12/13	WhiteMatter, BrainTissue, Brain
Cerebral Exterior		*CrblExt*	*14/15*	†
Cerebral White Matter		CrblWM	16/17	WhiteMatter, BrainTissue, Brain
Cerebrospinal Fluid		CSF	18	Brain
Hippocampus		Hc	19/20	DeepGreyMatter, BrainTissue, Brain
Inf. Lateral Ventricle		infLV	21/22	Ventricles, Brain
Lateral Ventricle		LV	23/24	Ventricles, Brain
Pallidum		Pa	25/26	DeepGreyMatter, BrainTissue, Brain
Putamen		Pu	27/28	DeepGreyMatter, BrainTissue, Brain
Thalamus Proper		Th	29/30	DeepGreyMatter, BrainTissue, Brain
Ventral DC		vDC	31/32	DeepGreyMatter, BrainTissue, Brain
Vessel		*Vsl*	*33/34*	†
Optic Chiasm		*OptC*	*35*	†
Cerebellar Vermal Lobules I-V		CVL1t5	36	DeepGreyMatter, BrainTissue, Brain
Cerebellar Vermal Lobules VI-VII		CVL6t7	37	DeepGreyMatter, BrainTissue, Brain
Cerebellar Vermal Lobules VIII-X		CVL8t10	38	DeepGreyMatter, BrainTissue, Brain
Basal Forebrain		BF	40/39	DeepGreyMatter, BrainTissue, Brain

† excluded as individual feature, neglectable size

Table 2. Cortical structures defined in NMM brain atlas. Structure names and how each individual structure was also considered as part of larger surrogate structures in this study. All cortical structures are outlined with a blue contour in the main manuscript.

**cortical** structure	abbreviation	ID (right/left)	Part of surrogate structure
anterior cingulate gyrus	ACgG	41/42	CorticalGreyMatter, BrainTissue, Brain
anterior insula	AIns	43/44	CorticalGreyMatter, BrainTissue, Brain
anterior orbital gyrus	AOrG	45/46	CorticalGreyMatter, BrainTissue, Brain
angular gyrus	AnG	47/48	CorticalGreyMatter, BrainTissue, Brain
calcarine cortex	Calc	49/50	CorticalGreyMatter, BrainTissue, Brain
central operculum	CO	51/52	CorticalGreyMatter, BrainTissue, Brain
cuneus	Cun	53/54	CorticalGreyMatter, BrainTissue, Brain
entorhinal area	Ent	55/56	CorticalGreyMatter, BrainTissue, Brain
frontal operculum	FO	57/58	CorticalGreyMatter, BrainTissue, Brain
frontal pole	FRP	59/60	CorticalGreyMatter, BrainTissue, Brain
fusiform gyrus	FuG	61/62	CorticalGreyMatter, BrainTissue, Brain
gyrus rectus	GRe	63/64	CorticalGreyMatter, BrainTissue, Brain
inferior occipital gyrus	IOG	65/66	CorticalGreyMatter, BrainTissue, Brain
inferior temporal gyrus	ITG	67/68	CorticalGreyMatter, BrainTissue, Brain
lingual gyrus	LiG	69/70	CorticalGreyMatter, BrainTissue, Brain
lateral orbital gyrus	LOrG	71/72	CorticalGreyMatter, BrainTissue, Brain
middle cingulate gyrus	MCgG	73/74	CorticalGreyMatter, BrainTissue, Brain
medial frontal cortex	MFC	75/76	CorticalGreyMatter, BrainTissue, Brain
middle frontal gyrus	MFG	77/78	CorticalGreyMatter, BrainTissue, Brain
middle occipital gyrus	MOG	79/80	CorticalGreyMatter, BrainTissue, Brain
medial orbital gyrus	MOrG	81/82	CorticalGreyMatter, BrainTissue, Brain
postcentral gyrus medial segment	MPoG	83/84	CorticalGreyMatter, BrainTissue, Brain
precentral gyrus medial segment	MPrG	85/86	CorticalGreyMatter, BrainTissue, Brain
superior frontal gyrus medial segment	MSFG	87/88	CorticalGreyMatter, BrainTissue, Brain
middle temporal gyrus	MTG	89/90	CorticalGreyMatter, BrainTissue, Brain
occipital pole	OCP	91/92	CorticalGreyMatter, BrainTissue, Brain
occipital fusiform gyrus	OFuG	93/94	CorticalGreyMatter, BrainTissue, Brain
opercular part of the inferior frontal gyrus	OpIFG	95/96	CorticalGreyMatter, BrainTissue, Brain
orbital part of the inferior frontal gyrus	OrIFG	97/98	CorticalGreyMatter, BrainTissue, Brain
posterior cingulate gyrus	PCgG	99/100	CorticalGreyMatter, BrainTissue, Brain
precuneus	PCu	101/102	CorticalGreyMatter, BrainTissue, Brain
parahippocampal gyrus	PHG	103/104	CorticalGreyMatter, BrainTissue, Brain
posterior insula	PIns	105/106	CorticalGreyMatter, BrainTissue, Brain
parietal operculum	PO	107/108	CorticalGreyMatter, BrainTissue, Brain
postcentral gyrus	PoG	109/110	CorticalGreyMatter, BrainTissue, Brain
posterior orbital gyrus	POrG	111/112	CorticalGreyMatter, BrainTissue, Brain
planum polare	PP	113/114	CorticalGreyMatter, BrainTissue, Brain
precentral gyrus	PrG	115/116	CorticalGreyMatter, BrainTissue, Brain
planum temporale	PT	117/118	CorticalGreyMatter, BrainTissue, Brain
subcallosal area	SCA	119/120	CorticalGreyMatter, BrainTissue, Brain
superior frontal gyrus	SFG	121/122	CorticalGreyMatter, BrainTissue, Brain
supplementary motor cortex	SMC	123/124	CorticalGreyMatter, BrainTissue, Brain
supramarginal gyrus	SMG	125/126	CorticalGreyMatter, BrainTissue, Brain
superior occipital gyrus	SOG	127/128	CorticalGreyMatter, BrainTissue, Brain
superior parietal lobule	SPL	129/130	CorticalGreyMatter, BrainTissue, Brain
superior temporal gyrus	STG	131/132	CorticalGreyMatter, BrainTissue, Brain
temporal pole	TMP	133/134	CorticalGreyMatter, BrainTissue, Brain
triangular part of the inferior frontal gyrus	TrIFG	135/136	CorticalGreyMatter, BrainTissue, Brain
transverse temporal gyrus	TTG	137/138	CorticalGreyMatter, BrainTissue, Brain

Table 3. Marshall Classification System. Based on and modified from Marshall et al. [1].

Marshall class	Description
1 Diffuse injury (DI) I	no visible intracranial pathological changes seen on CT
2 Diffuse injury II	cisterns are present with midline shift of 0–5 mm and/or: lesions densities present; no high or mixed density lesion >25 cm^3; may include bone fragments and foreign bodies*
3 Diffuse injury III (swelling)	cisterns compressed or absent with midline shift of 0–5 mm; no high or mixed density lesion >25 cm^3
4 Diffuse injury IV (shift)	midline shift >5 mm; no high or mixed density lesion > 25 cm^3
5 Evacuated mass lesion (EML)	any lesion surgically evacuated
6 Non-evacuated mass lesion (NEML)†	high or mixed density lesion >25 cm^3; not surgically evacuated

*As may be the case in depressed skull fractures.
†Merged into group 5 in data provided by Turku University Hospital.

Table 4. Glasgow Outcome Scale. Based on and modified from Jennett et al. [2, 3].

GOS	extended GOS	Description
1 (D)	1 (D)	*Dead*
2 (VS)	2 (VS)	*Vegetative state*: no evidence of meaningful responsiveness.
3 (SD)	3 (SD-)<br>4 (SD+)	*Severe disability*: conscious, but unable to live independently due to mental or physical disability.
4 (MD)	5 (MD-)<br>6 (MD+)	*Moderate disability*: able to live independently, limited ability to return to work or school.
5 (GR)	7 (GR-)<br>8 (GR+)	*Good recovery*: capacity to resume normal occupational and social activities, minor deficits possible.

Table 5. Acute-stage classification results (severe vs. moderate disability outcome). Classification results in % (6-fold cross-validation, 100 runs) obtained separating TBI patients with a severe disability from patients with moderate disability outcome based on structural volumes at the acute stage of the injury. Individual structures are sorted by effect size. Significant group differences indicated by + ($p < 0.05$) and ++ ($p < 0.001$), or “o” if not significant. Bonferroni corrected significance in parentheses. Individual features were classified using LDA, multiple features using RandomForest or SVM.

**Severe disability** (N = 13, Positives^{P}) vs. **Moderate disability** (N = 22, Negatives^{N}) **(cross-sectional analysis, acute stage)**								
structure	ACC (bACC)	SENS	SPEC	mean (SD) [mm^3]^P	mean (SD) [mm^3]^N	effect size (d)	p-value	sig. (corr.)
RandomForest (all cross-sectional features)	64 (58)	36	81					
SVM (all cross-sectional features)	64 (61)	45	76					
Gender (female = 0, male = 1)	66 (66)	69	64	0.3 (0.5)	0.6 (0.5)	0.673	0.06287	o (o)
Age	64 (63)	55	70	67.2 (15.9)	58.8 (10.0)	0.672	0.06331	o (o)
MarshallScore	57 (58)	62	55	3.9 (1.4)	3.0 (1.9)	0.510	0.15465	o (o)
GlasgowComaScale	43 (38)	20	57	12.0 (4.5)	12.6 (3.9)	0.155	0.66141	o (o)
InjurySeverity	38 (38)	35	40	2.8 (1.0)	2.8 (0.8)	0.083	0.81296	o (o)
DeepGreyMatter	67 (68)	73	63	164564.8 (20726.5)	180076.7 (20887.9)	0.745	0.04082	+ (o)
CorticalGreyMatter	67 (67)	66	68	459934.2 (63395.6)	494829.1 (39983.4)	0.701	0.05339	o (o)
BrainTissue	55 (55)	54	56	1130166.1 (102464.8)	1181045.4 (89378.0)	0.539	0.13272	o (o)
Ventricles	69 (67)	62	73	58766.8 (18808.4)	49592.9 (22303.5)	0.435	0.22267	o (o)
Brain	59 (58)	58	59	1191022.6 (110493.8)	1232890.5 (98415.8)	0.407	0.25346	o (o)
WhiteMatter	35 (34)	30	39	505667.1 (82222.8)	506139.5 (76357.7)	0.006	0.98638	o (o)
CerebellarVermalLobulesVIII-X	77 (78)	84	73	2659.5 (304.5)	3110.6 (394.1)	1.239	0.00121	+ (o)
AccumbensArea	76 (78)	84	71	460.0 (150.5)	609.9 (125.5)	1.110	0.00327	+ (o)
Amygdala	65 (67)	75	59	1795.5 (321.1)	2161.0 (378.9)	1.018	0.00642	+ (o)
ThalamusProper	60 (61)	63	59	11584.6 (1556.0)	13072.5 (1826.3)	0.859	0.01955	+ (o)
BrainStem	61 (62)	67	57	17417.2 (2275.5)	19416.8 (2417.5)	0.845	0.02144	+ (o)
BasalForebrain	63 (66)	77	55	548.7 (232.3)	773.2 (290.7)	0.829	0.02383	+ (o)
Hippocampus	64 (64)	62	66	5136.2 (1030.6)	6033.6 (1261.9)	0.759	0.03743	+ (o)
CerebellarVermalLobulesI-V	63 (64)	68	59	3471.9 (535.9)	3949.6 (704.4)	0.737	0.04283	+ (o)
VentralDC	61 (59)	54	64	8247.1 (1062.4)	8844.7 (880.6)	0.629	0.08149	o (o)
CerebellumExterior	61 (62)	66	57	94910.8 (12670.3)	103457.0 (14443.9)	0.618	0.08647	o (o)
Putamen	73 (74)	76	72	6066.7 (2492.2)	7087.0 (1586.9)	0.519	0.14721	o (o)
Pallidum	54 (54)	54	54	2801.2 (796.4)	3063.0 (442.4)	0.439	0.21786	o (o)
LateralVentricle	69 (68)	62	74	51162.3 (17123.4)	42726.9 (20449.5)	0.437	0.22046	o (o)
CerebellumWhiteMatter	65 (66)	69	63	36782.2 (7917.1)	33401.6 (8837.8)	0.397	0.26456	o (o)
InfLatVent	61 (61)	58	63	3131.4 (1360.4)	2649.1 (1182.9)	0.386	0.27817	o (o)
Caudate	63 (59)	43	74	7388.5 (3739.3)	6400.6 (1557.5)	0.384	0.28063	o (o)
CSF	52 (52)	51	52	2025.5 (719.9)	2194.0 (558.1)	0.271	0.44433	o (o)
3rdVentricle	60 (58)	54	63	2288.6 (814.2)	2076.9 (941.1)	0.236	0.50451	o (o)
4thVentricle	38 (36)	30	42	2184.5 (673.2)	2140.1 (576.0)	0.072	0.83738	o (o)
CerebralWhiteMatter	37 (36)	35	38	468884.8 (76690.5)	472738.0 (70658.1)	0.053	0.88084	o (o)
CerebellarVermalLobulesVI-VII	39 (39)	38	40	2076.6 (384.4)	2096.5 (498.0)	0.043	0.90263	o (o)
AsymmetryCerebellumWhiteMatter	72 (71)	69	73	19.2 (13.9)	9.6 (8.6)	0.886	0.01622	+ (o)
AsymmetryBrainTissue	71 (67)	54	81	5.2 (5.2)	2.4 (2.1)	0.799	0.02885	+ (o)
AsymmetryWhiteMatter	67 (64)	53	76	10.5 (8.5)	5.8 (5.1)	0.721	0.04713	+ (o)
AsymmetryAmygdala	74 (72)	65	80	16.9 (12.5)	8.5 (12.0)	0.697	0.05476	o (o)
AsymmetryDeepGreyMatter	68 (65)	53	76	3.9 (4.2)	1.9 (1.7)	0.695	0.05519	o (o)
AsymmetryBrain	69 (65)	51	80	4.0 (3.3)	2.3 (1.9)	0.653	0.07069	o (o)
AsymmetryCerebralWhiteMatter	62 (57)	40	75	10.2 (9.5)	5.7 (4.9)	0.646	0.07363	o (o)
AsymmetryPutamen	65 (58)	31	84	24.2 (44.3)	7.1 (8.3)	0.624	0.08343	o (o)
AsymmetryAllNonCortical†	67 (61)	39	83	262.0 (245.1)	164.4 (79.3)	0.607	0.09193	o (o)
AsymmetryLateralVentricle	66 (61)	39	82	31.5 (35.6)	16.7 (15.8)	0.590	0.10090	o (o)
AsymmetryVentricles	65 (60)	39	81	27.8 (32.4)	15.0 (14.7)	0.562	0.11767	o (o)
AsymmetryCerebellumExterior	69 (66)	54	77	4.8 (3.1)	3.2 (2.6)	0.561	0.11833	o (o)
AsymmetryAll†	60 (56)	39	73	1108.3 (369.6)	936.6 (282.8)	0.541	0.13122	o (o)
AsymmetryThalamusProper	65 (56)	19	92	14.2 (35.4)	3.2 (2.6)	0.511	0.15336	o (o)
AsymmetryPallidum	54 (47)	20	75	15.9 (32.6)	7.0 (4.7)	0.444	0.21275	o (o)
AsymmetryCaudate	65 (61)	44	77	17.3 (23.1)	9.9 (14.1)	0.412	0.24686	o (o)
AsymmetryHippocampus	58 (53)	36	71	16.2 (16.2)	11.7 (9.4)	0.363	0.30693	o (o)
AsymmetryAllCortical†	62 (58)	45	72	846.2 (160.7)	772.1 (229.7)	0.357	0.31432	o (o)
AsymmetryCorticalGreyMatter	62 (59)	49	69	4.9 (4.6)	3.6 (4.6)	0.296	0.40415	o (o)
AsymmetryVentralDC	60 (54)	32	77	9.5 (17.3)	6.4 (3.7)	0.284	0.42258	o (o)
AsymmetryBasalForebrain	47 (43)	30	57	37.3 (40.4)	31.1 (19.7)	0.213	0.54748	o (o)
AsymmetryAccumbensArea	53 (50)	38	62	23.4 (18.8)	20.1 (14.3)	0.202	0.56835	o (o)
AsymmetryInfLatVent	40 (41)	47	36	21.5 (20.5)	24.0 (26.0)	0.106	0.76275	o (o)

†: Sum of the AAIs of the individual structures.

Table 6. Acute-stage classification results (moderate vs. low disability outcome). Classification results in % (6-fold cross-validation, 100 runs) obtained separating TBI patients with a moderate disability from patients with low disability outcome based on structural volumes at the acute stage of the injury. Individual structures are sorted by effect size. Significant group differences indicated by + ($p < 0.05$) and ++ ($p < 0.001$), or "o" if not significant. Bonferroni corrected significance in parentheses. Individual features were classified using LDA, multiple features using RandomForest or SVM.

**Moderate disability** (N = 22, Positives^{P}) vs. **Low disability** (N = 32, Negatives^{N}) **(cross-sectional analysis, acute stage)**								
structure	ACC (bACC)	SENS	SPEC	mean (SD) [mm^3]^P	mean (SD) [mm^3]^N	effect size (d)	p-value	sig. (corr.)
RandomForest (all cross-sectional features)	61 (56)	32	80					
SVM (all cross-sectional features)	68 (64)	43	85					
MarshallScore	72 (68)	45	91	3.0 (1.9)	1.6 (1.1)	0.975	0.00091	++ (+)
InjurySeverity	69 (66)	55	78	2.8 (0.8)	2.3 (0.7)	0.666	0.01981	+ (o)
GlasgowComaScale	65 (60)	32	87	12.6 (3.9)	14.1 (2.2)	0.483	0.08720	o (o)
Age	58 (59)	62	56	58.8 (10.0)	61.1 (10.1)	0.223	0.42386	o (o)
Gender (female = 0, male = 1)	40 (38)	28	48	0.6 (0.5)	0.7 (0.5)	0.041	0.88325	o (o)
Ventricles	61 (61)	58	64	49592.9 (22303.5)	38131.9 (13556.0)	0.650	0.02268	+ (o)
CorticalGreyMatter	66 (67)	71	63	494829.1 (39983.4)	524318.2 (64687.5)	0.526	0.06297	o (o)
WhiteMatter	46 (44)	35	54	506139.5 (76357.7)	492768.2 (71084.9)	0.183	0.51279	o (o)
BrainTissue	49 (49)	49	48	1181045.4 (89378.0)	1198466.6 (135087.2)	0.147	0.59861	o (o)
DeepGreyMatter	42 (41)	38	44	180076.7 (20887.9)	181380.2 (22209.8)	0.060	0.82903	o (o)
Brain	40 (40)	39	40	1232890.5 (98415.8)	1238799.0 (138060.0)	0.048	0.86362	o (o)
InfLatVent	71 (69)	60	78	2649.1 (1182.9)	1913.3 (676.5)	0.804	0.00543	+ (o)
3rdVentricle	61 (61)	56	65	2076.9 (941.1)	1552.5 (617.3)	0.686	0.01660	+ (o)
AccumbensArea	58 (59)	64	53	609.9 (125.5)	696.3 (138.7)	0.647	0.02332	+ (o)
LateralVentricle	62 (61)	57	65	42726.9 (20449.5)	32704.8 (12459.3)	0.620	0.02953	+ (o)
Putamen	57 (56)	51	61	7087.0 (1586.9)	7849.7 (1338.7)	0.528	0.06207	o (o)
Hippocampus	54 (53)	49	58	6033.6 (1261.9)	6572.2 (873.5)	0.514	0.06913	o (o)
CerebellarVermalLobulesVIII-X	65 (65)	66	65	3110.6 (394.1)	2902.0 (420.4)	0.509	0.07185	o (o)
CerebellarVermalLobulesI-V	60 (61)	64	58	3949.6 (704.4)	4242.3 (699.1)	0.417	0.13786	o (o)
ThalamusProper	60 (61)	68	54	13072.5 (1826.3)	13832.8 (1845.3)	0.414	0.14127	o (o)
CerebellumWhiteMatter	56 (54)	45	64	33401.6 (8837.8)	30605.9 (8306.2)	0.328	0.24176	o (o)
4thVentricle	62 (62)	59	65	2140.1 (576.0)	1961.2 (526.9)	0.327	0.24336	o (o)
Amygdala	60 (60)	58	62	2161.0 (378.9)	2280.9 (380.7)	0.315	0.26007	o (o)
BasalForebrain	52 (51)	46	55	773.2 (290.7)	840.4 (233.9)	0.260	0.35230	o (o)
Caudate	51 (51)	50	51	6400.6 (1557.5)	6613.9 (1066.8)	0.166	0.55236	o (o)
CerebralWhiteMatter	43 (41)	33	50	472738.0 (70658.1)	462162.3 (67838.9)	0.153	0.58231	o (o)
Pallidum	49 (48)	41	54	3063.0 (442.4)	2990.8 (504.4)	0.151	0.58912	o (o)
CerebellarVermalLobulesVI-VII	48 (47)	43	52	2096.5 (498.0)	2150.0 (299.1)	0.137	0.62397	o (o)
CerebellumExterior	45 (44)	40	49	103457.0 (14443.9)	101795.8 (13850.8)	0.118	0.67217	o (o)
VentralDC	44 (44)	47	41	8844.7 (880.6)	8961.7 (1114.9)	0.114	0.68245	o (o)
CSF	45 (45)	42	47	2194.0 (558.1)	2140.6 (538.2)	0.098	0.72593	o (o)
BrainStem	44 (44)	47	42	19416.8 (2417.5)	19651.1 (2739.4)	0.090	0.74754	o (o)
AsymmetryVentralDC	62 (62)	64	61	6.4 (3.7)	4.2 (2.7)	0.696	0.01509	+ (o)
AsymmetryAccumbensArea	60 (59)	53	64	20.1 (14.3)	12.7 (10.2)	0.614	0.03097	+ (o)
AsymmetryAll†	63 (59)	37	80	936.6 (282.8)	826.6 (107.9)	0.555	0.05021	o (o)
AsymmetryAllCortical†	59 (56)	42	71	772.1 (229.7)	690.8 (88.0)	0.505	0.07390	o (o)
AsymmetryCerebralWhiteMatter	59 (57)	46	68	5.7 (4.9)	3.8 (2.8)	0.499	0.07738	o (o)
AsymmetryThalamusProper	61 (58)	43	74	3.2 (2.6)	2.2 (2.0)	0.476	0.09154	o (o)
AsymmetryCorticalGreyMatter	59 (55)	31	79	3.6 (4.6)	2.0 (2.3)	0.464	0.09964	o (o)
AsymmetryAllNonCortical†	58 (56)	47	65	164.4 (79.3)	135.8 (46.7)	0.463	0.10090	o (o)
AsymmetryPutamen	59 (55)	34	76	7.1 (8.3)	4.2 (4.8)	0.441	0.11713	o (o)
AsymmetryBrain	57 (55)	46	65	2.3 (1.9)	1.6 (1.5)	0.425	0.13126	o (o)
AsymmetryWhiteMatter	55 (53)	42	64	5.8 (5.1)	4.2 (3.1)	0.410	0.14463	o (o)
AsymmetryBasalForebrain	55 (54)	50	58	31.1 (19.7)	24.4 (17.1)	0.368	0.19000	o (o)
AsymmetryPallidum	58 (56)	47	65	7.0 (4.7)	5.5 (5.4)	0.282	0.31306	o (o)
AsymmetryCaudate	53 (50)	33	66	9.9 (14.1)	7.2 (6.0)	0.265	0.34372	o (o)
AsymmetryBrainTissue	50 (48)	39	57	2.4 (2.1)	1.9 (1.7)	0.264	0.34533	o (o)
AsymmetryHippocampus	48 (46)	36	57	11.7 (9.4)	9.9 (7.3)	0.223	0.42539	o (o)
AsymmetryInfLatVent	45 (44)	34	53	24.0 (26.0)	21.6 (17.7)	0.113	0.68420	o (o)
AsymmetryCerebellumWhiteMatter	46 (47)	53	41	9.6 (8.6)	10.5 (7.8)	0.103	0.71089	o (o)
AsymmetryLateralVentricle	45 (46)	54	38	16.7 (15.8)	18.2 (15.6)	0.096	0.73045	o (o)
AsymmetryAmygdala	45 (44)	34	53	8.5 (12.0)	7.7 (5.9)	0.090	0.74638	o (o)
AsymmetryCerebellumExterior	43 (45)	58	33	3.2 (2.6)	3.5 (3.7)	0.073	0.79238	o (o)
AsymmetryVentricles	42 (42)	42	41	15.0 (14.7)	15.4 (13.5)	0.033	0.90537	o (o)
AsymmetryDeepGreyMatter	41 (41)	41	41	1.9 (1.7)	2.0 (1.9)	0.020	0.94257	o (o)

†: Sum of the AAIs of the individual structures.

References

1. Marshall LF, Bowers Marshall S, Klauber MR, van Berkum Clark M, Eisenberg HM, Jane JA, et al. A new classification of head injury based on computerized tomography. Journal of Neurosurgery. 1991;75(1, Supplement):S14–S20.

2. Jennett B, Bond M. Assessment of Outcome after severe brain damage: A practical scale. The Lancet. 1975;306(7905):480–484.

3. Jennett B, Snoek J, Bond MR, Brooks N. Disability after severe head injury: observations on the use of the Glasgow Outcome Scale. Journal of Neurology, Neurosurgery, and Psychiatry. 1981;44:285–293.
